# When everything is not everywhere but species evolve: an alternative method to model adaptive properties of marine ecosystems

**DOI:** 10.1093/plankt/fbu078

**Published:** 2014-10-03

**Authors:** Boris Sauterey, Ben A. Ward, Michael J. Follows, Chris Bowler, David Claessen

**Affiliations:** 1 Environmental and Evolutionary Genomics Section, Institut De Biologie De L'Ecole Normale Supérieure (IBENS), CNRS UMR 8197, INSERM U1024, Ecole Normale Supérieure, 46 RUE D'ULM, 75005 Paris, France; 2 Environmental Research and Teaching Institute (CERES-ERTI), Ecole Normale Supérieure, 24 RUE Lhomond, 75005 Paris, France; 3 Laboratoire Des Sciences De L'Environnement Marin, Institut Universitaire Européen De La Mer, Place Nicolas Copernic, Plouzané, France; 4 Department of Earth, Atmospheric and Planetary Sciences, Massachusetts Institute of Technology, Cambridge, MA, USA

**Keywords:** phytoplankton, competition, trait-based adaptive strategies, adaptive dynamics, eco-evolutionary dynamics, trade-off, community, global circulation model

## Abstract

The functional and taxonomic biogeography of marine microbial systems reflects the current state of an evolving system. Current models of marine microbial systems and biogeochemical cycles do not reflect this fundamental organizing principle. Here, we investigate the evolutionary adaptive potential of marine microbial systems under environmental change and introduce explicit Darwinian adaptation into an ocean modelling framework, simulating evolving phytoplankton communities in space and time. To this end, we adopt tools from adaptive dynamics theory, evaluating the fitness of invading mutants over annual timescales, replacing the resident if a fitter mutant arises. Using the evolutionary framework, we examine how community assembly, specifically the emergence of phytoplankton cell size diversity, reflects the combined effects of bottom-up and top-down controls. When compared with a species-selection approach, based on the paradigm that “Everything is everywhere, but the environment selects”, we show that (i) the selected optimal trait values are similar; (ii) the patterns emerging from the adaptive model are more robust, but (iii) the two methods lead to different predictions in terms of emergent diversity. We demonstrate that explicitly evolutionary approaches to modelling marine microbial populations and functionality are feasible and practical in time-varying, space-resolving settings and provide a new tool for exploring evolutionary interactions on a range of timescales in the ocean.

## INTRODUCTION

Phytoplankton appeared 3 billion years ago (Hedges *et al*., 2001) and were the main actors responsible for the increase of oxygen in the atmosphere. Since then, they have become key regulators of the chemical composition of both the atmosphere and the oceans through processes such as photosynthesis (now contributing half of Earth's primary production) and the biological carbon pump (Falkowski *et al*., 1998; Field, 1998; Katz *et al*., 2004). Understanding the interaction between marine microbial systems and their environment is thus important. To realistically simulate the response of oceans and atmosphere to global changes, modelling approaches must therefore take microbial processes into account. Integrating the dynamics of marine communities and their physico-chemical environment (e.g. ocean vertical and horizontal turbulence, nutrient availability, light and temperature variations) in a practical way is a major objective. In this perspective, ocean circulation models are an efficient tool to produce realistic spatio-temporal structures as ecological “habitats” for plankton communities. However, coupling ecological models with highly complex physical models poses several challenges: the dimensionality, the number of parameters and the non-linearity of the system are high, as well as the required computational resources. Marine ecologists have therefore yet to set up efficient models of ecosystems as adaptive systems (i.e. whose fundamental characteristics may vary in response to changing environmental conditions), to identify and parameterize their different components (geo-physical parameters, phytoplankton and zooplankton populations, higher trophic levels) and their interactions.

The first nutrient–phytoplankton–zooplankton models (NPZ) were developed more than 65 years ago (Riley *et al*., 1949, and then Steele, 1958) but were coupled into three-dimensional global circulation models only much later (referred as GCMs) (Fasham *et al*., 1990; Sarmiento *et al*., 1993). In these studies, the plankton ecology was described by a set of three relatively simple equations for nutrients, phytoplankton and zooplankton dynamics, coupled by consumption, grazing and remineralization terms. The values of the biological parameters were chosen in order to make the model fit the observation data. The model ecosystem consisted of *ad hoc* ecological “black boxes”, useful to reproduce and describe observed patterns, but not adequate to provide details about the functional diversity characterizing marine microbial communities, nor to infer the functioning of the feedback loop between their characteristics and those of the environment. More recently, due to the increased amount of data about phytoplankton physiology from laboratory studies, some functional diversity has been resolved in models in order to take into account the complexity of ecological processes (Litchman *et al*., 2006; le Quéré *et al*., 2005). Finally, allometric trade-off-based approaches have been developed linking traits together and to phytoplankton cell size (Chisholm, 1992; Litchman and Klausmeier, 2008; Litchman *et al*., 2007, 2009; Verdy *et al*., 2009). Such allometry and trade-offs have been incorporated into community ecology approaches (Baird and Suthers, 2007; Banas, 2011; Follows *et al*., 2007; Ward *et al*., 2012), thereby introducing more realistic physiological and ecological mechanisms into the descriptions of plankton community dynamics.

Tackling the subject of functional diversity raises the issue of the multi-dimensionality of the traits space that defines the main characteristics of the considered organisms. In Follows *et al*. (Follows *et al*., 2007), the trait space defining phytoplankton is described as a multi-dimensional continuum and is explored by random seeding of the system by a large but finite number of species, each of them corresponding to a particular strategy (i.e. trait combination). An ecologically robust set of strategies then emerges from this initial diversity through species sorting (variation of the relative abundance of the species of the system) as a result of the imposed environmental conditions. Alternative approaches to explore the trait space can be random seeding spread over time as in Record *et al*. (Record *et al*., 2013) or discretization of the trait space as in Bruggeman and Kooijman (Bruggeman and Kooijman, 2007). Such methodologies are close to the posit that “everything is everywhere but the environment selects” (Becking, 1934) which is a keystone concept of the niche-assembly theory (for a review of the concept and its articulation in diverse fields in ecology, see de Wit and Bouvier, 2006). Population dynamics lead to the emergence of an ecologically “optimal” community from an initial species pool.

Everything-is-everywhere approaches implicitly hypothesize that the emerging community is equivalent to one that would result from the ecological and evolutionary history of the system even though the evolutionary processes themselves are not made explicit. It can, however, be argued that the evolutionary processes (mutation and selection) could affect the final outcome of the selection among the initial diversity and the final state of the adaptive process in particular because the everything-is-everywhere approach by definition is not appropriate to simulate the process of trait loss. In order to predict properly what the adaptive responses of marine systems would be, it is important to investigate this potential effect. Also, the everything-is-everywhere approach is not particularly economical in terms of computational demands due to the number of required species whose ecological dynamics must be solved in order to obtain robust patterns.

Eco-physiological trade-offs, when implemented in everything-is-everywhere models, can be used to conveniently tackle some fundamental ecological questions by predicting the optimal strategies for a given ecosystem, i.e. a successful set of traits, emerging from the biodiversity initially present through competitive exclusion. An important structuring variable that can be used to define such trade-offs in plankton communities is organism size (cell or body size). The causes of size diversity in plankton communities have been a recurrent subject of debate among marine ecologists. Empirical studies have revealed a strong allometric component to the variability of phytoplankton eco-physiological traits such as nutrient affinity, maximum growth rate and maximum uptake capacity (Litchman *et al*., 2007). Defining model parameters as a function of a single trait variable such as size enables one to considerably simplify the task of characterizing species (Baird and Suthers, 2007; Banas, 2011; Ward *et al*., 2012).

**Fig. 1.**
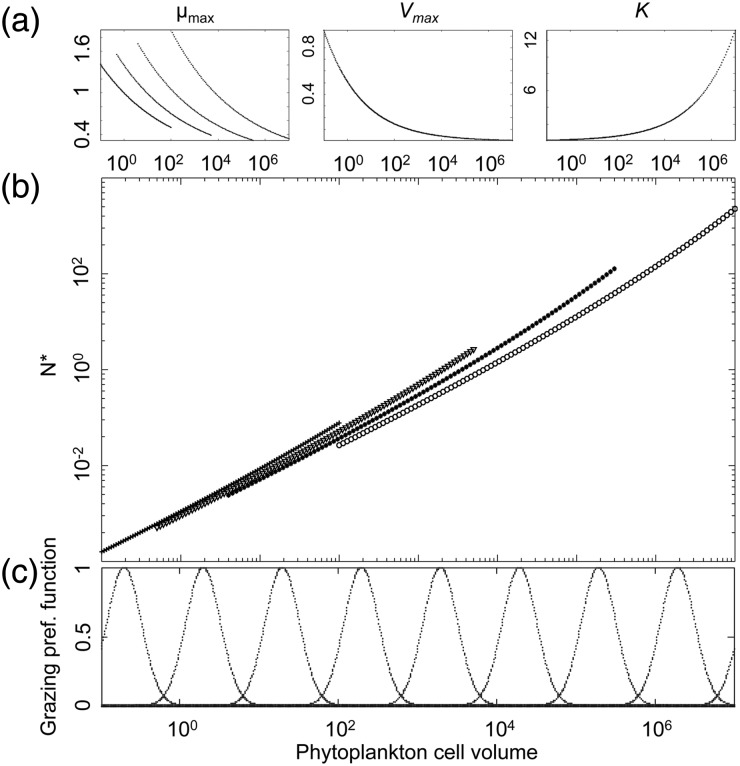
Size is a key physiological parameter: (**a**) log–log allometric relation linking cell volume to maximum photosynthetic rate (*µ*_max_), maximum nitrate uptake rate (*V*_max_) and nitrate half saturation constant (*K*_N_). Note here that the allometric exponent for *µ*_max_ varies among the four functional types, resulting in four curves. (**b**) Log–log relation between N* and the cell volumes and functional types of phytoplankton (crosses for *Synechococcus*, triangles for *Prochlorococcus*, plain circles for small eukaryotes and empty circle for diatoms). (**c**) Palatability of prey as a function of its cell volume.

Based on the observed allometric scaling of the different physiological parameters related to population growth, the classical model for phytoplankton population dynamics [the variable-internal-stores model (Droop, 1973; Grover, 1991)] predicts that the minimum required nutrient concentration N* increases with cell size (Box 1 and Fig. 1). The quantity N* is defined as the nutrient concentration below which a phytoplankton population declines and above which it grows. It is hence analogous to Tilman's R* in his resource competition theory (Tilman, 1977), and the prediction is thus that nutrient competition (i.e. bottom-up regulation) should favour the dominance of phytoplankton communities by the smallest cell sizes in accordance with the “principle of competitive exclusion” (Gause, 1934; Hardin, 1960). The strong size structure of phytoplankton communities, including coexisting small and large cell sizes, therefore raises an interesting ecological question first coined by Hutchinson (Hutchinson, 1961) as the “paradox of the plankton”: how do such diverse phytoplankton species, often limited by the same nutrients, coexist in ocean habitats (Chisholm, 1992; Falkowski and Woodhead, 1992)?

Box 1: Critical nutrient concentration N* in the variable-internal-stores model, without grazers

In the variable-internal-stores model initially described by Droop (Droop (1973) and Grover (Grover, 1991) and slightly modified by Flynn (Flynn, 2008), phytoplankton population growth (d*P*/d*t*) is decoupled from the extra-cellular nutrient concentration *N* by assuming that d*P/*d*t* is a function of the within-cell nutrient quota *Q*. The dynamics of *Q*, in turn, equal the difference between nutrient uptake and the conversion of cellular nutrient into growth:

(B1) $$\begin{array}{cc} & \frac{\text{d}Q}{\text{d}t}=\underset{\mathrm{Uptake}}{V_{\max}\cdot \left(\frac{Q_{\max}-Q}{Q_{\max}-Q_{\min}}\right)\cdot \frac{N}{N+K_{N}}}\\ & \;\;-\underset{\mathrm{Growth}}{\mu_{\max}\cdot \left(\frac{Q-Q_{\min}\;}{Q_{\max}-Q_{\min}\;}\right)\cdot Q} \end{array}$$

Here, *V*_max_ is the maximum nutrient uptake rate, *Q*_max_ and *Q*_min_ limit the values the internal reserves *Q* can reach, *K*_N_ is the half-saturation constant and *μ*_max_ the maximum growth rate. From the set of differential equations for *P*, *Q* and *N* [see the Supplementary data, Equations (S1), (S2) and (S3)], the equilibrium solution for the nutrient concentration N* can be derived as:

(B2) $$N^{*}=\frac{K_{N}\cdot \mu_{\max}\cdot (Q^{*}-Q_{\min})Q^{*}}{V_{\max}\cdot (Q_{\max}-Q^{*})-\mu_{\max}\cdot (Q^{*}-Q_{\min})\cdot Q^{*}}$$

In which Q* is the quota value at equilibrium:

(B3) $$Q^{*}=(Q_{\max}-Q_{\min})\;\cdot \frac{m}{\mu_{\max}}+Q_{\min}$$

where *m* is the size-independent death rate for phytoplankton.

N* is analogous to R* used by Tilman in his resource competition theory (Tilman, 1977): when competing for a single resource (*N*), the species with the smallest N* will outcompete all other species (in equilibrium). When the allometric scaling relations between model parameters and cell volume are known, we can hence compute N* as a function of cell size (Fig. 1b). Here, we assume that the allometric functions have the general form given by (some of these relations are plotted in Fig. 1a):

(B4) $$\log(x)=a+b\cdot \log\;(V_{\mathrm{Cell}})$$

where *x* is a given model parameter, *V*_cell_ is cell volume and *a* and *b* are allometric scaling constants. The allometric constant *a* for *μ*_max_ is modified by functional type, explaining the four *μ*_max_ and N* curves.

The basic prediction of competitive exclusion naturally assumes population dynamical equilibrium and the absence of predators. Many ecological factors may cause the coexistence of multiple competitors: non-equilibrium dynamics (Armstrong and McGehee, 1980); predation (Armstrong, 1994; Holt, 1977); and non-linearity of the ecological processes (Record *et al*., 2014). Furthermore, Cropp and Norbury (Cropp and Norbury, 2011) search through mathematical analysis the general conditions of stable species coexistence in food web models and describe it as a rather general case. Under the light of these studies, competitive exclusion appears as only one among many possible ecological scenarios. The consequences of some of these complicating factors, specifically grazing, were initially elucidated by studying such allometric NPZ models with size-dependent grazing without spatial structure (Baird and Suthers, 2007; Banas, 2011), and later in the context of a 3D global ocean model (Ward *et al*., 2012). In the latter study, both nutrient competition and predator–prey interactions are modelled in a size-dependent way (Fig. 1c and cf. Supplementary data, Section S2). Using the everything-is-everywhere approach to resolve the optimal strategies in terms of cell and body sizes of phytoplankton and zooplankton, Ward *et al*. (Ward *et al*., 2012) investigate the dual effect of top-down and bottom-up control on the possibility of ecologically stable coexistence of multiple size classes of phytoplankton and zooplankton, and the modification of this effect by local environmental conditions.

**Fig. 2.**
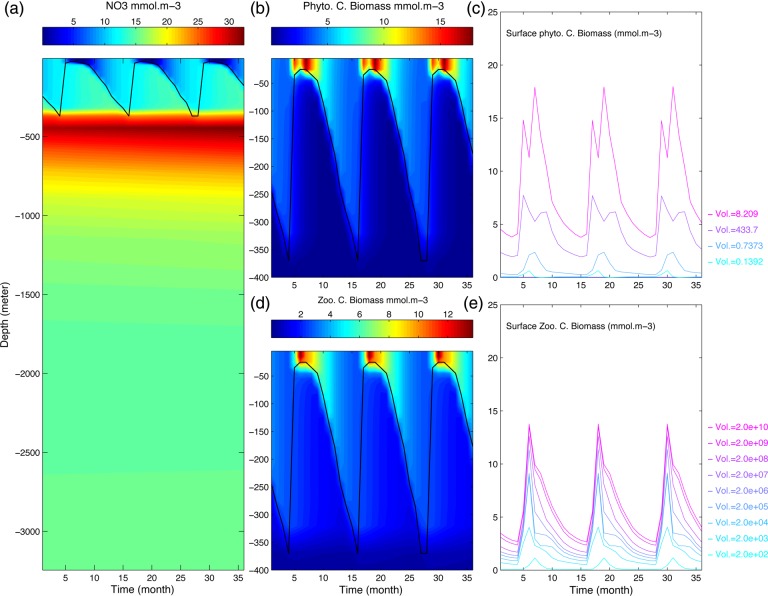
An illustration of the model dynamics, showing time series of key ecological variables over a 3-year period. (**a**) Nutrient (NO_3_) depth profile. (**b**) Depth profile (top 400 m only) of total phytoplankton carbon biomass (all species summed up). (**c**) Cumulative representation of surface layer biomass of 20 phytoplankton species. Colour indicates species cell size. Four species make up 99% of total biomass: one *Prochlorococcus*, two *Synechococcus* and one small eukaryote species, respectively. (**d)**: as (b) but for zooplankton. (**e)**: as (c) but for zooplankton.

One of the objectives of this study is to develop an alternative mechanistic approach for the exploration of the trait space in global ocean models: we propose to explicitly simulate the trait evolution of phytoplankton species in competition for shared resources and subject to shared predators, and the assembly of communities resulting from these interactions. Here, we present this approach in the context of an ocean model that represents a vertical water-column subject to seasonal forcing (Fig. 2a and Supplementary data, Figs S1a and S2). We introduced a simple evolutionary algorithm into a 1D version of the global (3D) ocean model used in Ward *et al*. (Ward *et al*., 2012). This algorithm periodically generates heritable intra-specific phenotypic variability within an initial pool of species, by introducing mutants whose invasive potential depends on their traits and the environmental conditions. It allows us to test the similarity of the communities selected when everything is everywhere, and when everything is not necessarily everywhere but species can evolve.

Our evolutionary model is inspired by the theoretical framework of adaptive dynamics (AD) (Metz *et al*., 1992). The idea of AD is to describe the trait evolution of a (group of) population(s), in an environment that is (at least partly) shaped by the trait-mediated impact of the population(s), assuming that evolution results from a succession of successful replacements of resident populations by invasive mutants. A characteristic of AD is that fitness is not considered as a fixed function of the characteristics of individuals. Instead, for a mutant with a given trait value, the invasion fitness is defined as the per capita growth rate of a negligibly small population of such mutants, in an ecosystem whose ecological dynamics have settled on its attractor. It is therefore a function of both the mutant's trait values, as well as the environmental conditions that depend on the resident populations' trait values. A mutant is considered successful if its invasion fitness is positive; its population will grow exponentially and it is generally assumed that it will replace the (ancestral) resident population through competitive exclusion. Once it has replaced its ancestor, the environmental conditions, and thus the fitness landscape (i.e. the invasion fitness of all mutants as a function of their traits), will be shaped by the traits of the newly installed population, now itself referred to as the resident. AD is therefore a practical method to represent fitness as a dynamical function of the traits of an ecosystem's evolving resident populations.

In our model, it is impossible to derive analytically the invasion fitness from the dynamical equations of the state variables (see Supplementary data, Section S2), due to the complexity of the dynamical system, its spatial structure and its time-varying forcing field (Fig. 2b and c). We thus have to explicitly model the mutants' dynamics and to compute their population growth rate. The evolutionary algorithm must be sufficiently simple to be easily implemented in an ocean model, but sufficiently sophisticated to allow (i) the introduction of mutants in the system when it has reached its current attractor; (ii) the computation of their invasion fitness; (iii) resident to be replaced by the successful mutant; and (iv) the new system to settle on its new attractor. We can then compare communities emerging from methodologies based on the everything-is-everywhere paradigm with communities resulting from the dynamics of evolving species. The questions behind our study are: (i) how is the predicted ecosystem influenced by the assumptions made about the source of the diversity (e.g. migration vs. mutation)? (ii) Does an assembly process with or without trait evolution select equivalent traits? And (iii) what is the influence of initial conditions on the emerging communities?

## METHODS

We extend the model presented by Ward *et al*. (Ward *et al*., 2012) by accounting for evolutionary dynamics of the phytoplankton species, using the AD framework to represent evolution. Below we briefly outline the model in terms of its ecological, physical/oceanographical and evolutionary components. A more complete description of the model can be found in Supplementary data, Section S2 and in Ward *et al.* (Ward *et al*., 2012).

### Ecological model

**Fig. 3.**
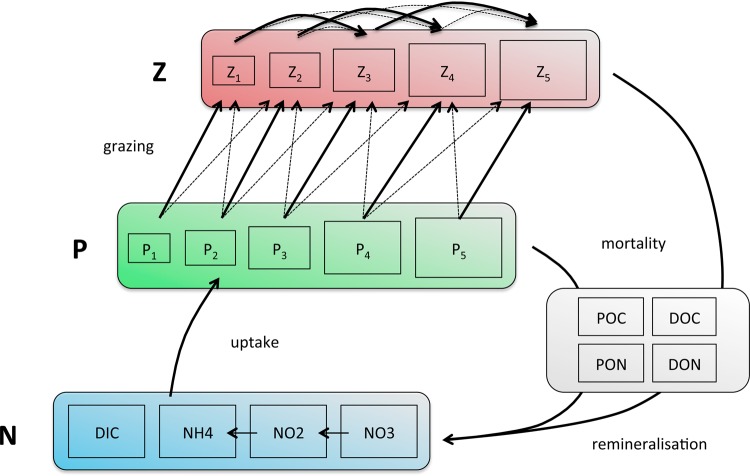
Compartments composing the ecological module of the model. *N* availability profile is set by the physics of the model and the consumption by P, which is itself regulated by Z through size-directed grazing. Loss is represented by a constant exponential mortality rate, and dead organic matter is then remineralized.

We use a plankton food-web model accounting for the dynamics of nutrients (three forms of nitrogen: NO_3_, $\text{N}{{\text{O}}_{2}}^{-}$, NH_4_) and a variable number of phytoplankton and zooplankton species (Fig. 3). The groups of species that occupy the same function in the trophic network (i.e. primary producers or grazers) are referred to as guilds. Each guild is assumed to contain multiple species, covering several orders of magnitude of organism size (cell volume or body size) and several trophic layers for the zooplankton guild.

As in many previous studies (among others Follows *et al*., 2007; Gregg *et al*., 2003; le Quéré *et al*., 2005; Moore *et al*., 2001), phytoplankton species are divided into four functional groups, *Prochlorococcus*, *Synechococcus*, small eukaryotes and diatoms. The idea that NPZ models—in which the functional diversity of the planktonic community is aggregated into two categories—are not adequate to mirror the complexity of its response to environmental variations was originally noted by Armstrong (Armstrong, 1994) and lead to the introduction into the models of plankton functional types. In our study, the functional groups are characterized by specific size ranges (Fig. 1), by specific allometric functions for the maximum growth rate (Fig. 1a) as well as by the impossibility for *Prochlorococcus* to use nitrate*.* Note here that the group-specific size dependence of the growth rate explains the difference between the four N* curves in Fig. 1b.

Zooplankton are predators of both phytoplankton and smaller zooplankton (the latter is referred to as intra-guild predation). Following Ward *et al*. (Ward *et al*., 2012), we assume a Holling type 3 functional response for the grazing interaction. In addition to grazing mortality, phytoplankton and zooplankton are subject to a constant background mortality rate. Once the living organic biomass is transformed into organic detritus by grazing loss or mortality, it is then remineralized.

The following eco-physiological traits depend on phytoplankton cell size: maximum nutrient uptake rate (*V*_max_), half-saturation concentration (*K*), cellular carbon content, minimum and maximum nitrogen quota (*Q*_min_, *Q*_max_), maximum growth (*µ*_max_) and sinking rate. For zooplankton, the maximum prey ingestion rate is a function of body size. For any pair of potential prey and predator, the grazing efficiency is described by a log-normal function of the predator-to-prey cell size ratio centred around an optimal value [cf. Supplementary data, Section S2 and Equation (S11)]. Equations and parameters are fully described in Supplementary data, Section S2 and in Ward *et al*. (Ward *et al*., 2012).

### Physics of the water column

The depth-structured model consists of a 3275-m deep water-column divided into 35 levels (cf. Ward *et al*., 2012). Forcing fields (light, temperature, winds) are used to annually constrain the vertical mixing of the water column (Supplementary data, Figs S1a and S2). This forcing corresponds to environmental conditions in a high latitude ocean as described by Dale *et al*. (Dale *et al*., 1999) for the Norwegian Sea: high annual variations of the light and temperature conditions as well as the level of stratification of the water column. The seasonality of the forcing of the abiotic conditions shapes the habitat of plankton populations and therefore their population dynamics. For instance, annual variation of the vertical movements in the water column can result through mixing processes in the periodic transfer a significant proportion of the phytoplankton biomass outside of the euphotic zone and thus limit their growth, or conversely in significant injection of nutrients into the euphotic zone.

The dynamics of the biomass of both phytoplankton and zooplankton modelled in the water column is similar to observed systems in the Norwegian Sea (see Supplementary data, Figs S1b and S2). Two main phases can be characterized: (i) a winter period (between December and March) during which the lack of light is limiting for phytoplankton growth, and (ii) bloom and post-bloom periods (respectively, April to June and July to November) during which the living biomass increases due to stratification of the water column and an increase in light intensity, followed by a decrease in phytoplankton when nutrients are exhausted and grazing dominates.

### Evolutionary model

The novel part of this study is the implementation of AD of the phytoplankton populations into a 1D configuration of the MIT GCM, structured both in time (annual periodicity) and in space (depth). The evolving trait we chose is cell volume. Since phytoplankton are defined in terms of cell volume and functional type, the whole set of size-dependent traits is affected by the evolution of cell volume. We use the term “residents” for the phytoplankton species that are present at a given time. Their evolving traits are denoted by $X_{j}$ with *j*=1, …, *k*, where *k* is the current number of residents.

Our evolutionary algorithm periodically generates two hypothetical mutants for each resident phytoplankton species; one is slightly bigger (10% of cell volume), and the other slightly (10%) smaller. The mutation step size is assumed to be constant (we have verified that the value of the mutation step size does not qualitatively affect the results). The introduced mutants are assumed to be very rare which allows the further assumption that the mutant population does not impact the environment (resources, grazers), while it does experience it. After an initial transient, the mutant population will start growing exponentially with a constant rate (on a year-to-year basis) once it has reached its stable relative depth profile (of biomass and quota). Biologically, a mutation would appear highly localized in space, and therefore the transient can be considerable before the mutant population reaches the stable relative depth profile. Invasion fitness is computed as the mutant's asymptotic exponential growth rate. In order to shortcut the long transient, we assume that a mutant population's initial vertical biomass and quota profiles are identical to that of the ancestral resident. Biologically, this modelling trick can be interpreted as assuming that (i) the mutant spreads quickly across the water column; (ii) the mutant's relative depth profile is similar to the resident's which seems likely due to the similarity in trait value; (iii) the transient to the relative depth profile is rapid enough for the assumption of rareness of the mutant population to remain valid until it reaches the stable distribution.

Once introduced into the water column, the population dynamics of the mutants are computed during a single year. Over this period, mutant invasion fitness is computed (see below). The evolving trait of mutant *i* of resident *j* is denoted by $Y_{\;j,i}$. A mutant's invasion fitness depends on its evolving trait but also on the traits of all the residents because the residents contribute to shape the environmental conditions (resource and zooplankton densities). Invasion fitness *s* can hence be written as (Geritz *et al*., 1998; Metz *et al*., 1996):

(1) $$s_{\;j,i}=f(Y_{\;j,i}\;|\;[X_{1},\ldots ,X_{K}])$$

Conversely, the population dynamics (and hence fitness) of resident species is not affected by the presence of the mutants in the system, due to their rareness. The system's dynamics are assumed to be on the attractor when the mutants are introduced and so the dynamics of the resident population is supposed to be on an annual limit cycle. Hence, the annual resident's population growth rate is supposed to be zero.

In the simulations, invasion fitness is computed as follows:

(2) $$s_{\;j,i}=\log\left(\frac{B_{\;ji}(t+1)}{B_{\;ji}(t)}\right)$$

where *B_ji_*(t) is the total biomass of mutant population *i* of resident population *j* (summed over the water column) at the time of introduction and *B_ji_*(*t* + 1) is its biomass 1 year later. For each phytoplankton species, the evolutionary algorithm computes the invasion fitness of each mutant and of the resident. Note that according to the definition of invasion fitness, a mutant with the same trait as the resident has fitness equal to zero (implying steady population dynamics), whereas a slightly smaller mutant or a slightly larger mutant may have negative or positive invasion fitness. In practice, the system does not always reach its ecological attractor, and the resident fitness *s_j_* is thus close to but not equal to zero. We therefore assume that the condition required for a mutant to replace a resident population is to have a higher invasion fitness than the resident, while the second mutant has a fitness lower than the resident (i.e. directional selection). However, for the sake of clarity, we keep to using the terms “positive” and “negative” invasion fitness. Three scenarios can thus be encountered: (a) directional selection: one of the mutants has positive invasion fitness, whereas the second has a negative one; (b) stabilizing selection: both mutants have negative fitness; (c) disruptive selection: both mutants have positive fitness.

In the case of scenario (a), which is the most common one, we assume that “invasion implies replacement” meaning that the mutant with a positive fitness replaces the resident population. This corresponds to what Geritz *et al*. (Geritz *et al*., 2002) call “attractor inheritance” and hold under fairly mild conditions if the mutation is sufficiently small for the invading mutant to be sufficiently similar to the former resident [which forbids cases of attractor switching and “the resident strikes back” phenomena described by Mylius and Diekmann (1995)]. In our algorithm, the trait of the successful mutant is attributed to the old resident population, its spatial distribution remaining the same. During a period of 2 years, the system is then allowed to converge to the new ecological attractor corresponding to the trait values of the new residents, before a new set of mutants is introduced. The full mutation–invasion–replacement–transient cycle thus lasts 3 years and shapes the AD through repetition.

Such directional selection eventually results in evolutionary convergence towards a so-called singular strategy in trait space, which is either a fitness maximum (scenario b) or a fitness minimum (scenario c). In scenario (b), the resident is an evolutionarily stable strategy (ESS). The fact that the population has converged to this ESS through directional selection further implies that it is also convergence stable; it is therefore referred to as a continuously stable strategy (CSS, Eshel, 1983). In scenario (c), the resident is an evolutionary branching point (EBP; a convergence stable strategy with disruptive selection). Both mutants can invade (and probably replace) the resident, resulting in divergence of two new, sister resident populations (Geritz *et al*., 1998). In our system, all the converging singularities are of the CSS type (scenario b). Disruptive singularities do exist, but they are repelling and hence not EBP (see Supplementary data, Section S3).

To summarize, our method is valid if (i) the mutation step is sufficiently small to assume attractor inheritance when selection is directional, (ii) a 2-year period is sufficient for the new resident's system to be sufficiently close to its attractor for fitness evaluation to be reliable, and (iii) the system's attractor is annually periodic. Verification of the first and second hypotheses can be found in Supplementary data, Section S3, and we know that the third is true due to the strong forcing that the ecological dynamics is submitted to.

### Simulation protocol

Each simulation is seeded with a variable number of phytoplankton species (8, 20, 40 or 80). Each species is first allocated to one of the four functional types (*Prochlorococcus*, *Synechococcus*, small eukaryote or diatom). A cell volume is then drawn randomly from the size range that is specific for the functional type. The system is also seeded with nine species of zooplankton whose body sizes are spread in such a way that the range of grazed phytoplankton size covers the entire range of possible phytoplankton sizes. The resulting grazing background is log-periodic with regions of phytoplankton size submitted to a low grazing pressure, referred to as grazing refuges, and others for which the grazing pressure is high (cf. Fig. 1c). It should be noted, however, that the realized grazing background depends on the population dynamics of the zooplankton species (assumed to be identical in Fig. 1c). The initial depth profile of these species is uniformly low (6 × 10^−9^ mmol C m^−3^). The initial environmental conditions correspond to winter (January) temperature and light conditions.

Each simulation consists of two phases: the “ecological phase” without evolution and the “eco-evolutionary phase” with evolution. During the first, ecological phase species sorting occurs among the initially present species with their fixed traits. The population dynamics are determined by the specific characteristics of each species, i.e. by their size and functional type, and eventually converge to an attractor with 1-year periodic dynamics. This attractor defines a community of coexisting species (phytoplankton and zooplankton) that can be considered as a set of the best competitors among the initially randomized set of strategies; the remaining species go extinct. It is also characterized by a specific environment in terms of the grazing profile, nitrate availability, etc. The convergence towards this ecological attractor is relatively quick (about 10–20 years). In our simulations, this period lasts 100 years to ensure that we obtain the asymptotic dynamics.

During the second eco-evolutionary phase, the phytoplankton traits evolve. The ecological interactions are thus repeatedly modified by trait evolution within the community resulting in an eco-evolutionary transient. The characteristics of the system do not change solely due to changes in the relative abundances of the species, but also through their adaptive response to environmental conditions. The eco-evolutionary transient is usually longer than the ecological one. We set, after numerous test simulations, the duration of this phase to 250 years to make sure that the second attractor is reached. We also verify at the end of each simulation that the system is on its attractor. The second community thus obtained is composed of a set of evolutionarily attracting strategies (CSS or EBP). At the end of the run, we check whether selection is stabilizing (CSS) or disruptive (EBP). In all our runs, the final community consists exclusively of CSS.

The succession of the two phases allows us to compare the characteristics of the two emerging communities and the evolutionary stability of the first one. The set of four seeding conditions also allows testing the effect on the two attractors of the initial level of trait diversity in the system.

### Indicators and statistics

In order to characterize the two phases, we focus on a small number of characteristics. We measure biodiversity in two different ways. First, species richness *R* is defined as the number of species with abundance above an arbitrary extinction threshold. (In this implementation of the model, species abundance cannot reach values below an artificial threshold (10^−15^ mmol C m^−3^), an assumption that mimics the effect of immigration from the surrounding water column).

Second, we use the Shannon index to measure numerical diversity, which characterizes how uniformly biomass is distributed among the present (non-extinct) strategies:

(3) $$S=\frac{\sum_{i=1}^{N}p_{i}\;\log(\;p_{i})}{\log(N)}$$

here *N* is the number of discrete size classes (we arbitrarily fix it to 100), and *p_i_* is the proportion of the total biomass belonging to organisms of the size class *i*. Note that a given size class may contain more than one species with very similar sizes.

Third, we use the Canberra distance (denoted by CD) to measure the distance between two cell-volume distributions among the discrete size classes (comparing before and after evolution, or between two runs). We compare the biomass in each size class *i* within each of the two distributions *x* and *y*. The CD is computed as:

(4) $$\text{CD}(x,y)=\overset{N}{\underset{i=1}{\sum }}\frac{|x_{i}-y_{i}|}{|x_{i}|+|y_{i}|}$$

In order to estimate the intra-group variability between cell size structures of the different communities, we measure the mean square distance among them based on the CD:

(5) $$\text{MSD}=\frac{\sum_{k=1}^{K}\text{CD}\;{\left(x_{k},\hat{x}\right)}^{2}}{K}$$

with *K* being the number of simulations for each seeding conditions (24 here) and $\hat{x}$ the mean cell size distribution within this group.

### Fitness landscape

For a single example run, we compute the full fitness landscapes at the end of the two phases. To do so, we introduced for each functional type exhibiting at least one resident in the eco-evolutionary attractor of the simulation a large number of mutants (200) in order to cover an almost continuous and broad size spectrum, and computed the mutants' invasion fitness.

## RESULTS

**Fig. 4.**
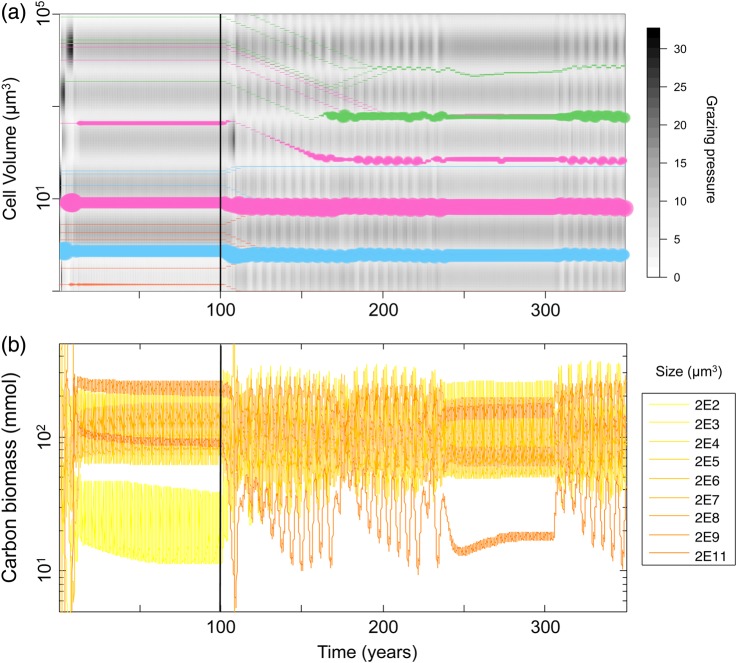
Illustrative example of model dynamics. The vertical line at *t* = 100 separates phase 1 (fixed traits) from phase 2 (phytoplankton trait evolution). (**a**) Each coloured line is a phytoplankton species; its width represents its log-abundance; the ordinate represents the trait value (cell volume). The smallest line width represents quasi-extinct species maintained by immigration. Colours represent functional groups: orange = *Prochlorococcus*, cyan = *Synechococcus*, magenta = small eukaryotes and green = diatoms. The background grey-shaded map represents the grazing pressure depending on the zooplankton dynamics and the size of the phytoplankton. (**b**) Population dynamics of the zooplankton species.

We will describe separately the two phases of the experimental protocol, focusing first on the patterns emerging from one illustrative simulation seeded with 20 phytoplankton species (5 for each of the functional types) with randomly generated cell sizes (Fig. 4a), and then looking at the whole set of runs for each seeding condition.

### The ecological phase

During this first phase, the convergence of the system towards its attractor is relatively fast: about 20 years or so. Figure 4a shows the rapid emergence of four dominant species among which two are particularly abundant (a *Synechococcus* and “small eukaryote” species). As the population dynamics converge towards annual limit cycles, all their competitors go to quasi-extinction, shaping an ecologically stable community. At the same time, zooplankton populations also tend towards their asymptotic cycle resulting in a periodically stable grazing background (Fig. 4b). Note that the emerging phytoplankton species are characterized by a large diversity of cell volume, as well as by a variety of functional types. Nevertheless, they share one characteristic: their cell sizes correspond to what we will refer to as “grazing refuges”, i.e. cell sizes for which the predation pressure is relatively low (clear areas in the background of Fig. 4a), suggesting that predator avoidance is a key factor regarding species success.

**Fig. 5.**
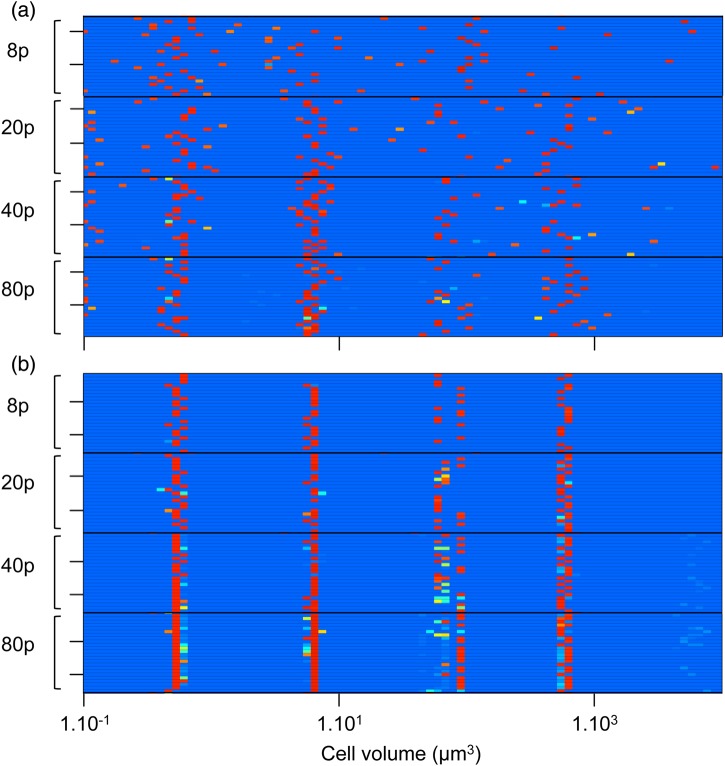
Heat map of the phytoplankton species size distribution (the functional types are merged here) at the end of the first phase (*t* = 100 years) in (**a**) and the second phase (*t* = 350 years) in (**b**) in each of the 24 simulations, for each of the four seeding conditions (8, 20, 40 or 80 phytoplankton species). The colour of each grid represents the number of individuals with the corresponding size in the system. From light blue for absent or negligible sizes (i.e. maintained only by migrations) to red for abundant species. Similar species are grouped into the same size interval.

**Fig. 6.**
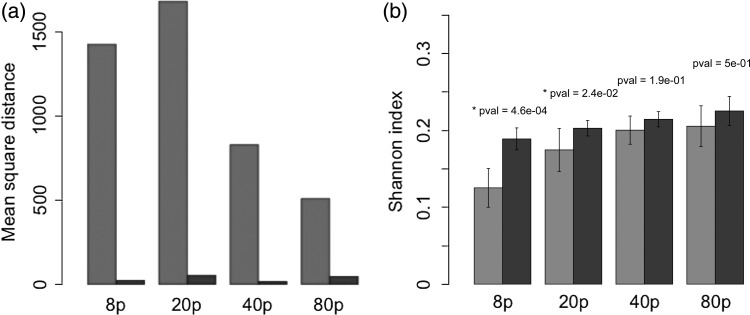
(**a**) Average Canberra distance between the mean distribution and the individual simulations, for each attractor (light for *t* = 100 years, dark for the *t* = 350 years), and for each seeding condition (8, 20, 40 or 80 initial phytoplankton species). (**b**) The mean Shannon index for the same groups.

Looking at the whole set of runs (Fig. 5a), we can first note that the communities emerging from the species sorting during the first phase are highly diverse: the example (Fig. 4a, *t* = 100) is not representative of what we observe in the other runs (Fig. 5a). First, in terms of cell size composition of the community, a clear size pattern cannot be identified in Fig. 5a for low seeding resolution (8 and 20 phytoplankton species), and the corresponding intra-group mean square distances are particularly high (Fig. 6a). However, at higher seeding resolution (40 and 80 species), the average MSDs are lower (Fig. 6a) and the selected cell sizes appear to be centred around four values (Fig. 5a), corresponding to the emergence of four dominant species.

It thus appears that the ecological system we model is characterized by a set of four “niches” corresponding to four optimal competitors that can coexist and that can exclude all the other species by competition when they are in the system. But when (some of) these four species are not in the system, phytoplankton populations with diverse cell sizes are allowed to settle in, as in the example shown in Fig. 4. In addition, it appears that these optimal species minimize the grazing pressure they are submitted to by having cell volumes corresponding to grazing refuges set up in the grazing background of our simulations, confirming the idea that the sensitivity to grazing is as expected an important characteristic determining the competitive ability of a species (Supplementary data, Fig. S6).

Finally, it appears that the distribution of biomass across size classes is highly dependent on the initial conditions. Figure 6b shows the average Shannon index value for each seeding condition. The figure shows that a higher seeding resolution results in a more even biomass distribution among species. The effect is saturating for high seeding resolutions and can be described by a power law (after log-linearizing the data, a linear regression shows a positive effect of the seeding resolution with a *P*-value of 1.35 × 10^−6^).

### The eco-evolutionary phase

Let us come back to the example run shown in Fig. 4a. The starting point of the second phase of the simulation is the ecological attractor described above. The evolution of the traits disturbs the ecological state of the system (thus ecologically but not evolutionarily stable): some species decline (the *Prochlorococcus*), some species slightly adjust their cell volume and remain stable (the two previously highly abundant species), some switch from one grazing refuge to another and some new species evolutionarily emerge and settle as new dominant species (diatoms). Because the ecological interactions in the system are being constantly renewed by the AD, the transient is much longer than in the first period: the new eco-evolutionary attractor is reached after almost 100 years. This new attractor is also composed of four species, whose sizes are grazing refuges; however, these cell sizes are not equivalent to those present at *t* = 100.

**Fig. 7.**
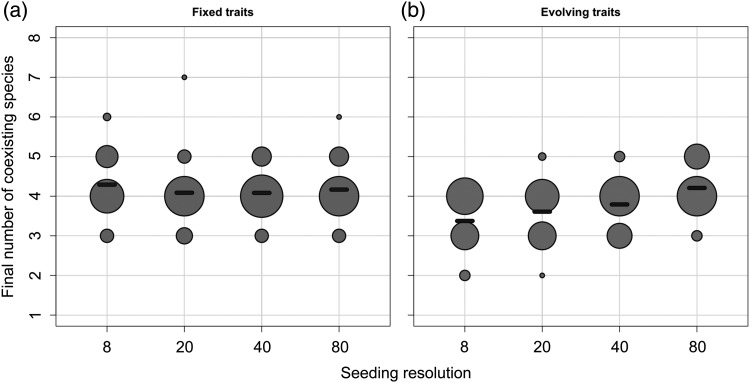
Number of viable populations (excluding quasi-extinct species, i.e. species whose abundance is below the arbitrary threshold of 1×10^−^^3^ mmol C of total biomass) at the end of the two phases and for each seeding condition. The size of a dot reflects the number of simulations with the considered final number of species. The black dashes represent the average of each group.

Conversely to the attractor of the ecological phase in the example run (Fig. 4a), it appears that the state of the system of our example at *t* = 350 properly reflects the general patterns observed in the whole set of ecological runs (cf. Fig. 5a). In general, the states of the system at *t* = 350 are much more conserved among runs, and among groups than they were at *t* = 100 (and thus less dependent on the initial conditions): intra-group MSD is much lower (Fig. 6a), as well as the intra- and inter-group variability of the Shannon index (Fig. 6b) and the variability of the number of species the community is composed of (Fig. 7).

The characteristics of the attractors of the eco-evolutionary phase are furthermore very similar to what has been observed in the ecological phase with 80 species seeding. More precisely, for each seeding condition, the majority of the runs at *t* = 350 displays an evolutionarily stable community of four species whose cell sizes are extremely conserved (Figs 5b and 6a), and correspond to the size ranges identified as grazing refuges, given the grazing background (note that the phytoplankton cell size values corresponding to grazing refuges depend on the zooplankton sizes that are fixed and on their abundances that are dynamic). These four cell sizes are similar to the four optimal competitors identified in the ecological phase for the runs with 80 species (Fig. 5a).

In the eco-evolutionary phase, the Shannon index is generally high (Fig. 6b), and the positive effect of seeding resolution, though significant (*P* = 1.34 × 10^−4^), is much weaker. As a consequence, when seeding resolution is low (i.e. with 8 and 20 initial species), the Shannon index increases significantly during the eco-evolutionary phase (the Kruskal–Wallis test, respective *P*-values are 4.6 × 10^−4^ and 2.4 × 10^−2^). Interestingly, the opposite pattern can be observed regarding species richness (Fig. 7). Although there is no significant effect of the seeding resolution on the number of species in the ecological attractors (still relatively high), the number of species in the eco-evolutionary attractors increases with seeding resolution, converging towards values similar to those of the ecological attractor (a linear regression gives a significant positive effect with a *P*-value = 4.2 × 10^−2^, Fig. 7b). Hence, when seeding resolution is low (eight initial species), species richness decreases during the eco-evolutionary phase (the Kruskal–Wallis test, *P*-value of 5.6 × 10^−4^).

### Periodicity of the system

Supplementary data, Figure S7a and b shows details of the population dynamics of both phytoplankton and zooplankton species for the last 15 years of the two phases. It is clear that the system is annually periodic between *t* = 85 and *t* = 100 years in the ecological phase (and as expected phytoplankton and zooplankton are in phase opposition), as a direct consequence of the physical forcing. The dynamics of the system in the eco-evolutionary phase between *t* = 335 and *t* = 350 years is more complex: a 6-year periodical component is superimposed on the annual forcing. This 6-year period corresponds to an evolutionary oscillation of the traits of the dominant species (Supplementary data, Fig. S7c) and is hence an artefact of our chosen time schedule of mutant invasion and resident replacement, as well as our assumption of a fixed mutation step size.

## DISCUSSION

**Fig. 8.**
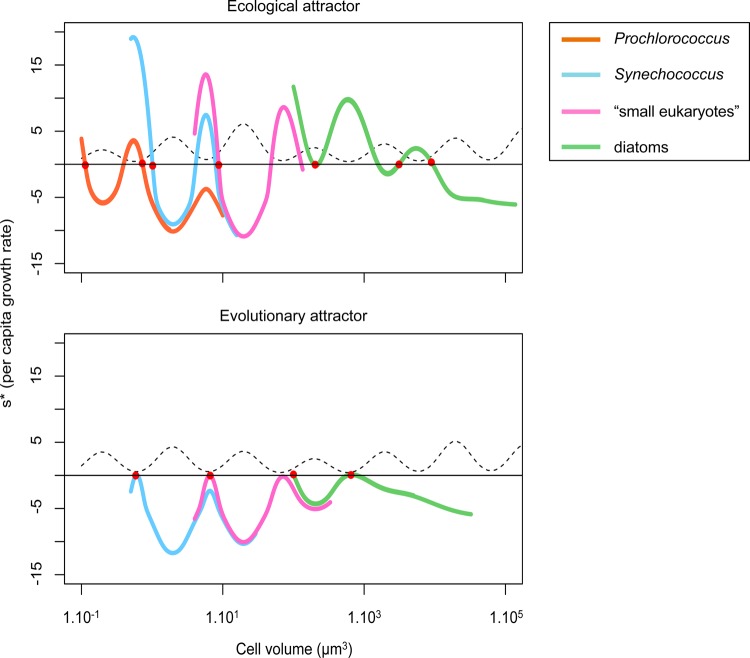
Fitness landscapes (invasion fitness as a function of cell volume) corresponding to an attractor with species whose traits are fixed on the top, and with adapting species on the bottom. Colours represent functional groups: orange = *Prochlorococcus*, cyan = *Synechococcus*, magenta = small eukaryotes and green = diatoms. The dotted line represents the grazing pressure that the phytoplankton are submitted to as a function of their size. The cell sizes of the coexisting species are represented with red dots.

We have introduced AD into an ecological model with a very complex spatial and temporal structure. Based on the MIT general circulation model, our model represents a vertical water column of 3000 m in which the physical forcing in terms of temperature oscillation, vertical mixing and diffusion are parameterized to represent conditions in the Norwegian Sea. The ecological model itself is highly dimensional, representing an ecosystem of nine zooplankton species, between 8 and 80 phytoplankton species, 4 different nutrients as well as 4 compartments of dead-organic matter. Ignoring the spatial structure, the ecological model itself requires between 59 and 275 ordinary differential equations. This is far more complex than common applications of AD that are usually studied in simple and unstructured population or community models, or in models with a single structuring dimension [i.e. either spatial structure (Haller *et al*., 2013) or size structure (Claessen and Dieckmann, 2002)]. Not surprisingly, the ecological dynamics in such a complex and high-dimensional model are complex as well (cf. Fig. 2). Yet in spite of this complexity, introducing AD has enabled us to elucidate the ecological and evolutionary dynamics of the phytoplankton species in terms of basic ecological concepts such as bottom-up vs. top-down regulation. A number of our main conclusions can be illustrated in an intuitive way by considering the shape of the fitness landscape in the two scenarios we consider: without evolution (Fig. 8, top panel) and with evolution (Fig. 8, bottom panel). Figure 8 shows for each functional type the shape of the invasion fitness as a function of cell size. We can recognize the effect of the two main constraints in the shape of the curves: (i) the grazing pressure results in oscillations (fitness is higher where grazing is lower; compare the dotted curve of grazing pressure); and (ii) each functional type shows an overall decrease of the invasion fitness with cell volume which results from the relative size disadvantage of bigger cells in terms of nutrient uptake and requirements. The top panel of Fig. 8 is an example in which seven species coexist after the non-evolutionary phase. These species all have zero fitness [which follows from the definition of invasion fitness (Metz *et al*., 1992)]. None of the species is CSS; although two diatoms are relatively close to the same fitness maximum. One diatom is even at a fitness minimum. The bottom panel of the same figure clearly illustrates why evolution can reduce species richness: all remaining species are CSS (situated at fitness maxima). Whereas in the top panel, two species can share the same “hill” in the fitness landscape; once the hilltops are reached, this is no longer possible. We also observe that an entire functional group (*Prochlorococcus*) has disappeared; the evolution of the smallest possible *Synechococcus* size has made persistence of any *Prochlorococcus* size impossible, which is empirically supported (Flombaum *et al*., 2013). Below we give a more detailed interpretation of the effect of evolution on the emergent diversity, and in particular of the effect of the initial species richness on the resulting patterns.

### Periodic grazing background

Our results are strongly influenced by two basic ecological constraints that result directly from our model assumptions: nutrient competition favours small cell sizes; a periodic grazing background favours cell sizes in grazing refuges. The observed communities at the end of our simulations contain species that perform well under these two basic constraints. Whereas the first assumption is emerging from the size dependence of the biological rates as documented in the literature (Chisholm, 1992; Litchman and Klausmeier, 2007; Litchman *et al*., 2009), the second is imposed to the system by the number and body size of zooplankton species, as well as by the shape of the grazing efficiency function. Whereas predation is generally size dependent (Cohen *et al*., 1993; Fuchs and Franks, 2010) and its efficiency may be described by a log-normal function of the predator-to-prey biovolume ratio (Hansen *et al*., 1994, 1997; Kiørboe, 2008), several arguments can be forwarded for a grazing background smoother than the log-periodic one we choose to use in this study. First, increasing the number of phytoplankton species of different body sizes will gradually smooth out the periodic grazing background. Second, each zooplankton population may be (and, in the real world, most certainly is) characterized by an intra-specific size variation resulting from inter-individual differences and also from a variation of an individual's cell size during its development. The size range thus exhibited by a specific grazer species will result in a corresponding range of optimally grazed prey size, again smoothing the grazing background (Claessen *et al*., 2002). *In situ*, the actual shape of a local grazing background is difficult to assess but would be a most interesting empirical element in this context. We choose here to hypothesize a log-periodic grazing background which generated fixed CSSs and simplified the analysis of the results. Theoretically, however, the shape of the grazing background (periodic or smooth) is likely to influence the outcome of the ecological and evolutionary dynamics. Indeed, in reality, it is almost certainly a result of the predator–prey coevolution. However, a full implementation of a co-evolving predator–prey system is beyond the scope of this article but is the focus of a further study (Sauterey *et al*., in preparation).

### High initial richness: “everything is everywhere”

In our scenario with the maximum initial level of species richness (80 species, Fig. 4a), the phytoplankton trait space is practically fully covered, and so we are close to the “everything is everywhere” situation: all possible strategies compete with each other at any given time (a scenario very similar to the approach in Ward *et al.* (Ward *et al.*, 2012)). In the absence of evolution, species sorting results in a robust pattern among runs of the dominance of the phytoplankton community by four sizes (cf. Figs 4a and 5a). The repeatability of this pattern suggests that these four trait values are ESSs (Smith and Price, 1973) in the sense that once they occupy the system they cannot be beaten by any other strategy. More precisely, the community of four phytoplankton species resembles an evolutionary stable coalition (Geritz *et al*., 1997). The evolutionary stability of this phytoplankton community is confirmed by the fact that, in the second phase of the simulations, the evolutionary process does not alter the trait composition of the phytoplankton community. Metz *et al*. (Metz *et al*., 1992) show that an ESS, despite its evolutionary stability, is not necessarily an evolutionary attractor, and so not necessarily likely to be the end result of the evolutionary process. In our case, the adaptive process almost systematically converges to the above-mentioned ESSs—whether they are present in the initial community or not (Figs 3a and 4b). We can thus consider that these four strategies are not only ESS, but are also evolutionarily attractive. We conclude that the final result of natural selection in our model ecosystem does not depend on whether the species sorting occurs on populations with fixed or evolving traits, as long as “everything is everywhere”, i.e. as long as the initial species diversity is sufficiently high.

**Fig. 9.**
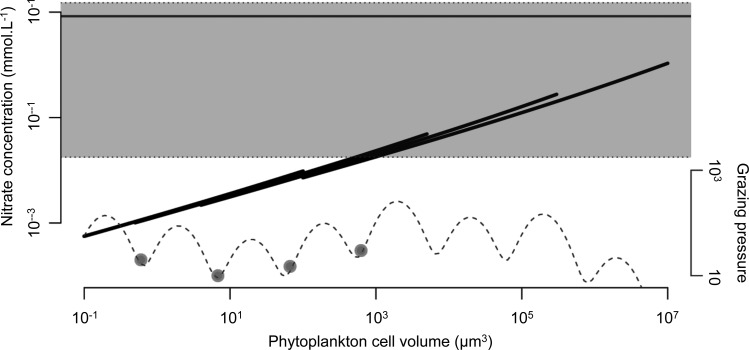
The large curves are the ideal N* curves, without considering light or temperature limitation (the realized N* may thus be higher) as a function of cell size, for the four functional types. The dotted curves represent the grazing pressure applied on each size. The horizontal line is the annual mean *N* concentration conditions a phytoplankton cell meet during the last year of simulation, and the grey-shaded area is delimited by its min and max. The dots mark the cell sizes of the CSSs.

This first analysis also allows hypotheses on the co-existence from an eco-evolutionary point of view. We showed that, as a consequence of the existence of grazing refuge in the grazing background, the ability of phytoplankton species to avoid predation is fundamental regarding their competitive ability. Altough this prediction depends on the existence of grazing refuges, and therefore on the design of the model, it is particularly consistent with the hypotheses presented by Smetacek (Smetacek, 2012) who argues, based on observations, that the predator–prey coevolution is very important regarding the structure of the marine ecosystem. However, we also noted that not all the grazing refuges are CSSs, but only the four smallest ones. This can be explained by considering a second factor: nutrient limitation. Figure 9 shows the N* and the grazing background as a function of phytoplankton cell size and at the same time the *N* surface concentration corresponding to a community comprising the four CSSs. All the CSSs have N* lower than or equal to the minimum *N* concentration (note, however, that in order to compare these values, we assume that the light and temperature limitations we removed from the N* calculation are negligible). The other grazing refuges correspond to N* values much higher than the minimum *N* concentration, meaning that these species met conditions for which their intrinsic population death rate is higher than their growth rate during an important part of the year, and thus are unlikely to maintain themselves in the system. Hence, though the non-linearity of the nutrient supply forbids any simple investigation, it appears that due to the log-periodic shape of the grazing background, top-down controls narrow down the possibility of coexisting species to grazing refuges, and that bottom-up control allows only the smallest of them to persist. In our system, the emerging community progressively shapes the environmental conditions, in particular in terms of nutrient availability. As the number of species in the system increases, and therefore the phytoplankton organic biomass, these conditions are depleted to a point that no supplementary species can invade or be maintained in the ecosystem (Armstrong, 1994; Ward *et al*., 2012). Note here that ecological interactions other than top-down regulation by grazers, such as host-parasites/pathogens, are expected to have the same structuring effect on phytoplankton population. Viruses especially appear to be an important component of phytoplankton population dynamics (Brussaard, 2004; Fuhrman, 1999; Suttle, 2007). Exploration of the effect of viruses on the structure of plankton communities could therefore be an interesting lead to follow by completing the range of ecological interactions taken into account in models of microbial communities. While this study confirms that dual effect of top-down and bottom-up controls generate ecologically stable coexistence of multiple phytoplankton size classes, we also show that this coexistence is evolutionarily stable and attracting. However, this evolutionary convergence occurs for a rather artificial grazing context given the imposed log-periodic shape of the grazing background and the fact that zooplankton cannot adapt in response to prey evolution. Modelling predator–prey coevolution could give different results. Previous theoretical studies suggest that for similar systems, predator–prey coevolution could under certain conditions favour evolutionary emergence of diversity from monomorphic populations (Brown and Vincent, 1992; Dercole and Rinaldi, 2008; Dercole *et al*., 2003; Ito and Ikegami, 2006; Loeuille and Loreau, 2005).

### Low initial richness: “everything is not everywhere”

The probability of the presence of the four ESSs in the initial set of species increases with the level of initial trait diversity. Hence, at low initial diversity, the probability of the emergence of all four ESSs (or at least species sufficiently close to the four ESS strategies) is low in the ecological phase. A system that does not initially include species close to all optimal strategies allows for the persistence of “non-optimal” species, i.e. species that would be outcompeted by the nearest ESS species, if the latter are present (Figs 4a and 8). In such cases, selective pressure is released by the lack of optimal competitors: a single predation refuge may be occupied by none, one or more non-optimal species. This fact is apparently contradictory with the “competitive exclusion principle” (Gause, 1934; Hardin, 1960) which formalized the idea that if one resource is shared by more than one species, only the best competitor persists, excluding the others. However, as explained by Armstrong (Armstrong, 1994), two different populations sharing one resource can coexist, even in equilibrium, if their populations are regulated by two specific grazers (or other specific density-dependent loss terms). In our model, when one of the four ESSs is absent, the corresponding predation refuge may be occupied by two species (one for each specific grazer). Ultimately, this means that the number of species in the system may in fact exceed the number of evolutionarily viable predation refuges, resulting in the coexistence of more than four phytoplankton species. High levels of species richness can thus occur (Figs 7 and 8). Yet overall, due to the fact that few of these species are sufficiently well adapted to the environmental conditions (especially to grazing) to reach high levels of abundance, the Shannon index is usually rather low for these cases of high species richness (Fig. 6b). To summarize, in the ecological phase, the selection of a specific strategy relies on its presence in the initial trait composition but also on the ecological interactions between the initially present species (including zooplankton). Species sorting results in the emergence of the best viable strategies (yet not necessarily ESSs) within the initial pool of species. On average, for low initial trait diversity, the emergence of non-optimal strategies is frequent, often resulting in high species richness but low Shannon diversity.

With evolution (the eco-evolutionary phase), the selection of a specific strategy does not only rely on the initial trait composition and the resulting ecological interactions, but also on whether the strategy is evolutionarily attractive. We have underlined the fact that all the ESSs of the system are also CSSs, and so the probability of evolutionary emergence of the optimal size values is high whether the initial trait richness is high or not. Due to this almost systematic evolutionary convergence towards unbeatable strategies, non-optimal species are not maintained in the second phase. This explains the fact that in terms of species richness, the communities emerging from the ecological phase are on average richer than communities emerging from the eco-evolutionary phase for low seeding resolutions (Fig. 7). However, whereas the sizes selected by species sorting among evolving species are systematically CSSs, all the CSSs are not necessarily always simultaneously present (especially in the lowest seeding conditions), stressing the dependence of the adaptive process on the initial state of the system. This dependence can be explained by the fact that the evolutionary optima are local and are surrounded by evolutionarily repelling regions (i.e. the grazers' optimal prey sizes): if no species initially has a strategy located between two adjacent repelling regions, the CSS cannot be reached through a gradual evolutionary process.

The sensitivity to initial conditions can also be observed in a slight variation in size in the third grazing refuge (∼300 µm^3^). In the different runs, one of two different sizes dominates in this grazing refuge (Fig. 5). The smallest systematically corresponds to the functional type referred to as “small eukaryote”, the second to a diatom. These two sizes have their own disadvantages: the lower boundary of the diatom size range is above the “valley” of the grazing background, whereas a small eukaryote is inherently characterized by a higher N* than a diatom of an equivalent size. However, Fig. 8 shows that when a diatom dominates the niche, potential eukaryote invaders have slightly negative invasion fitness. This is confirmed by looking at the eco-evolutionary trajectories: small eukaryotes are allowed to invade only if diatoms cannot evolve into this grazing refuge, depending on the initial condition (i.e. if all the diatoms initially have sizes superior to the upper grazing peak and are therefore evolutionarily “stuck”). The probability that this scenario occurs decreases with the number of diatom species in the system, as can be observed in Fig. 5b.

### Computational costs and robustness of the results

We have shown that the two different approaches (i.e. the everything-is-everywhere approach and the evolutionary approach) allow estimations of the “optimal” phytoplankton strategies for the modelled habitat. However, the robustness of the results is stronger in the evolutionary approach. The lower dependence on the initial conditions of the eco-evolutionary method has two major consequences. First, the required number of species is much lower. Without evolution, 80 initial phytoplankton species are necessary to obtain the four CSSs with a 50% probability in a single simulation (Fig. 5), whereas with evolution only 20 species are required. Note, however, that three times as many tracers are needed due to periodical mutant introduction. Second, we show that the emerging patterns are much more conserved among runs thanks to the repeatability of the evolutionary convergence (and hence much less reliant on the initial randomization of traits). Without evolution and with 80 phytoplankton species, the intra-group MSD is 10 times higher than with evolution and only 20 species.

### The two phases as analogies for adaptive responses of ecosystems

The adaptive response of ecosystems to environmental conditions is a process comprising many mechanisms. We argue that these mechanisms could be described as belonging to two conceptual categories. The two phases and their differences described in this study can be seen as representatives of these two components of the general process of modification of the characteristics of an ecosystem.

The first process could be defined as the ecological adaptive potential of an ecosystem: the change in an ecosystem's properties results from the variation of the relative abundance of its species. These ecological mechanisms occur on an ecological time scale and so evolving trait dynamics are thus (usually) negligible. The trait diversity therefore relies on the ecological properties of the system, such as the connectivity to adjacent systems and their environmental conditions (Wilson, 1992), its ecological history (Robinson and Edgemon, 1988) or phenotypic plasticity (Charmantier *et al*., 2008). For instance, in our model, the high diversity that characterizes marine microbial systems, and their openness, justify the use of randomization to seed the system.

We refer to the second process as the evolutionary adaptive potential of ecosystems. In contrast with the first process, this kind of ecosystem adaptation occurs on an evolutionary timescale: the modification of the ecosystem's characteristics is not only due to the variations of the abundances of its species but also to their individual adaptation to environmental variations. The adaptive response is therefore also influenced by parameters that determine the evolutionary dynamics: mutation rates, genetic constraints (a given species is characterized by a range of trait values it can take), the speed at which the fitness landscape varies, etc. Different theories, including AD and population genetics, describe such evolutionary trajectories, although usually of much simpler systems than the one considered in the present study.

The apparent separation between ecological and evolutionary adaptive potential is, however, a rather artificial consequence of the way the concerned scientific fields grew independently. We emphasize in this study the entanglement of ecology and evolution: the evolution of the characteristics of an organism depends directly on its ecology, whereas in turn the ecological characteristics of a system constantly vary as a result of the individual evolution of the species living in it. In real conditions, we could thus expect adaptive responses of ecosystems to belong to a continuum in which ecological and evolutionary responses vary in proportion depending on the considered system and timescale. The outcome of the interaction between ecology and evolution is particularly of interest in order to understand the response of ecosystems to environmental variations, especially for systems comprising organisms with short generation times such as plankton, in which ecological and evolutionary timescales are overlapping (Carroll *et al*., 2007; Hairston *et al*., 2005). Previous efforts to tackle the long-term response of marine systems to environmental changes in three-dimensional ocean simulations have not considered any potential evolutionary changes. These attempts, from “ocean biogeochemical models” by Maier-Reimer and Hasselmann (Maier-Reimer and Hasselmann, 1987) and Najjar *et al*. (Najjar *et al*., 1992) to the more recent “dynamic green ocean models” (le Quéré *et al*., 2005), all consider fixed ecological properties of marine communities in a varying environment. Interestingly, “everything-is-everywhere” approaches are able to solve this problem by considering that marine systems are characterized by a quasi-infinite potential of ecological adaptation. One aspect is nonetheless still missing: evolutionary adaptation is absent from the model, and thus it is impossible to verify the evolutionary attractiveness of the emerging communities. But as described in this study, by making explicit both the ecological and the evolutionary processes, a coupled approach allows the simultaneous investigation of all the components of the adaptive response of an ecosystem.

### Emerging perspectives

The whole eco-evolutionary feedback loop can thus be studied: the effect of the environment on trait evolution of interacting species, the resulting modification of the ecological interaction between these species and thus the variation of their relative abundance, and ultimately the consequences of the modified ecology on the environmental conditions.

Real-time coupling between ecology and trait evolution allows study of the dynamical properties of the fitness landscape of a system as a product of the evolution of the ecological interactions: it allows investigation of the mechanisms that link the evolution of one species to the dynamics of the fitness landscape for all the species that interact directly or indirectly with it. This approach is thus a convenient tool to study any model of co-evolution (between prey and predator, competitors, host and parasites and so on).

However, a crucial dimension of the eco-evolutionary dynamics remains absent from our model: although the evolutionary algorithm allows a successful evaluation of the direction of the evolution and identification of attracting singularities in the space of traits in terms of CSS or EBP, it is unable to model what happens in the case of evolutionary branching, and therefore to describe speciation. This question is of particular importance when considering predator–prey coevolution, which has already been described as potentially resulting in a cascade of evolutionary branching events (Loeuille and Loreau, 2005). Practically, the number of state variables, and therefore of the number of species, is usually constrained in models such as the GCMs and the addition of species is therefore problematic. This problem is apparent in the case of sympatric speciation (local disruptive selection; EBP) but also in the case of allopatric divergence in the case of a spatially structured eco-evolutionary model: a specific mutant could (and most of the time will) have positive invasion fitness in some region of the ocean and a negative one in others. Such a case should lead to the splitting of a resident population into two sister populations, geographically separated from each other. In such scenarios, our evolutionary algorithm would theoretically be able to identify the possibility of branching, but it is incapable of modelling its consequences. An additional, more conceptual, challenge in such situations is the definition of invasion dynamics and of invasion fitness. On large spatial scales, our assumption of using an instantly stable distribution of mutant biomass and quota (by copying the resident's distribution) becomes less and less valid.

Alternative approaches should therefore be considered. Agent-based models are often used to model processes of evolutionary branching (Dieckmann and Doebeli, 1999; Doebeli and Dieckmann, 2003) and have recently been applied to global ocean circulation models (Clark *et al*., 2013; Daines *et al*., 2014). The important computational demand that usually characterizes these models (expected to be particularly high in a 3D GCM) can be strongly limited through the use of meta-agents: many individuals of similar properties are lumped together in “super-individuals” (Rose *et al*., 1993; Scheffer *et al*., 1995). These increasingly used models could allow explicit implementation of evolutionary dynamics along the lines of AD to study the process of evolutionary branching. Another strategy is to use evolutionary models with trait-structured populations (characterized by a finite number of state variables), which have been used to approximate and mimic the behaviour of individual-based models (Champagnat *et al*., 2006; Perthame and Gauduchon, 2010; Sauterey *et al*., in preparation). In addition, there is no practical constraint to the implementation of these latter evolutionary models into GCMs. They could therefore be satisfactory tools to study spatially structured phytoplankton–zooplankton co-evolution.

The implementation of such models into 3D ocean circulation models would permit an extension of the work presented here to the effect of spatial structures on the interactions between ecology and evolution. For example, what is the effect of the spatial variation of environmental conditions on the evolution of the traits of metapopulations? This question is particularly important in marine systems where both the amplitude of the variations of environmental conditions and the connectivity between regions are highly spatially variable and greatly impact the ecology of the species living there.

## SUPPLEMENTARY DATA

Supplementary data can be found online at http://plankt.oxfordjournals.org.

## FUNDING

This research was conducted as part of the Phytback Project, which is supported by the Agence Nationale pour la Recherche. M.J.F. is grateful for support from the Gordon and Betty Moore Foundation (Grant #3778). C.B. acknowledges funding from the EU Micro B3 project and ERC Diatomite project. Funding to pay the Open Access publication charges for this article was provided by the Agence Nationale de la Recherche via grant PHYTBACK (ANR-10-BLAN-7109).

## Supplementary Material

Supplementary Data
