# Supplementary material for: When everything is not everywhere but species evolve: an alternative method to model adaptive properties of marine ecosystems
Source: J Plankton Res. 2014 Oct 3;37(1):28–47. doi: 10.1093/plankt/fbu078 (PMC4378374; doi:10.1093/plankt/fbu078)
Supplement: Supplementary Data [file supp_37_1_28__index.html]

When everything is not everywhere but species evolve: an alternative method to model adaptive properties of marine ecosystems — When everything is not everywhere but species evolve: an alternative method to model adaptive properties of marine ecosystems — Supplementary Data 

# When everything is not everywhere but species evolve: an alternative method to model adaptive properties of marine ecosystems

## Supplementary Data

Supplementary Data

**Files in this Data Supplement:**

- Supplementary Data - Docx file
