# Supplementary material for: When everything is not everywhere but species evolve: an alternative method to model adaptive properties of marine ecosystems
Source: J Plankton Res. 2014 Oct 3;37(1):28–47. doi: 10.1093/plankt/fbu078 (PMC4378374; doi:10.1093/plankt/fbu078)
Supplement: Supplementary Data [file supp_fbu078_fbu078supp.docx]

Supplementary material

**Supplementary material 1: *Spatially unstructured variable internal stores model***

Light and temperature limitation are here neglected:

| $\frac{dP}{dt}= \mu_{max}.\left( \frac{Q-Q_{min}}{Q_{max}-Q_{min}} \right).P-m.P$ | (S1) |
| --- | --- |
| \| $\frac{dQ}{dt}= V_{max}.\left( \frac{Q_{max}-Q}{Q_{max}-Q_{min}} \right).\frac{N}{N+K_{N}} -\mu_{max}.\left( \frac{Q-Q_{min}}{Q_{max}-Q_{min}} \right).Q$ \| \| --- \| | (S2) |
| \| $\frac{dN}{dt}= {I(t) - V}_{max}.\left( \frac{Q_{max}-Q}{Q_{max}-Q_{min}} \right).\frac{N}{N+K}.P$ \| \| --- \| | (S3) |

P is phytoplankton biomass, *Q* is the within-cell nutrient quota, *N* is the extra-cellular nutrient concentration. *I* is the nutrient import term depending on the environmental forcing, *V_max_* is the maximum uptake rate, *Q_max_* and *Q_min_* the maximum and minimum values *Q* can reach, *K* is the half saturation constant, µ*_max_* the maximum growth rate. This model is close to an variable-internal-stores model from (Droop 1973; Grover 1991) slightly modified by (Flynn 2008).

**Supplementary material 2: *Spatially structured model:***

Dynamical equations of the 1D model for inorganic nutrients concentration

| \| $\frac{\partial\left[ N_{i} \right]}{\partial t}= -\nabla\cdot\left( \mathbf{u}\left[ N_{\boldsymbol{i}} \right] \right)+\nabla\cdot\left( \mathbf{K}\nabla\left[ N_{i} \right] \right)- \sum_{j=1}^{J} \left[ B_{C_{j}} \right]V_{i,j}+R_{i}$ \| \| --- \| | (S4) |
| --- | --- | --- |

for phytoplankton carbon and nitrogen biomass concentration

| \| $\frac{\partial\left[ B_{i_{j}} \right]}{\partial t}= \nabla\cdot\left( \mathbf{K}\nabla\left[ B_{i_{j}} \right] \right)+ {\left[ B_{i_{j}} \right]V}_{i,j}-\frac{\left[ B_{i_{j}} \right]}{\left[ B_{C_{j}} \right]}\sum_{z=1}^{Z} \left[ B_{C_{z}} \right]G_{C,j,z}-\left[ B_{i_{j}} \right]m+\frac{\partial\left[ B_{i_{j}} \right]w_{j}}{\partial k}$ \| \| --- \| | (S5) |
| --- | --- | --- |

And for zooplankton carbon biomass

| \| $\frac{\partial\left[ B_{C_{z}} \right]}{\partial t}= \nabla\cdot\left( \mathbf{K}\nabla\left[ B_{C_{z}} \right] \right)+\left[ B_{C_{z}} \right]\sum_{j=i}^{J} G_{C,j,z}-\left[ B_{C_{z}} \right]m+\frac{\partial\left[ B_{C_{z}} \right]w_{z}}{\partial k}$ \| \| --- \| | (S6) |
| --- | --- | --- |

where

| **K** | Mixing coefficients from the physical model |
| --- | --- |
| *t* | Time |
| *i* | Subscript denoting element type |
| *j* | Subscript denoting phytoplankton species |
| *z* | Subscript denoting zooplankton species |
| *V_i,j_* | Uptake rate of the nutrient *i* by the phytoplankton *j* |
| *G_C,j,z_* | Grazing rate for predator *z* on the carbon biomass of the phytoplankton *j* |
| *m* | Linear mortality rate |
| *w* | Sinking rate |
| *S* | Additional sources and sinks |
| *k* | Depth |

- In each point of the grid the net carbon uptake by phytoplankton *j* can be described by

| \| $V_{C,j} =\mu_{max}\cdot\gamma_{N}\gamma_{T}\gamma_{L}$ \| \| --- \| | (S7) |
| --- | --- | --- |

where γ*_N_* is the limitation factor related to the internal nitrogen status which is defined as:

| \| $\gamma_{N} =\frac{Q_{N,j}-Q_{min,N,j}}{Q_{max,N,j}- Q_{min,N,j}}$ \| \| --- \| | (S8) |
| --- | --- | --- |

and $\gamma_{T}$ and $\gamma_{L}$ are respectively temperature and light dependence as defined in Ward et al (2012). The nitrogen quota *Q_N_* itself is defined as $\frac{\left[ B_{i_{j}} \right]}{\left[ B_{C_{j}} \right]}$, decreases due to photosynthesis according to (A7) and increase through nitrogen uptake as follow:

| \| $V_{N,j} =V_{max,i,j}\cdot\frac{N_{i}}{N_{i}+K_{i,j}}\cdot\left( \frac{Q_{max,N,j}-Q_{N,j}}{Q_{max,N,j}- Q_{min,N,j}} \right)$ \| \| --- \| | (S9) |
| --- | --- | --- |

Where $N_{i}$ are the different inorganic sources of nitrogen.

- The grazing rate of a predator *z* on a prey *j* is described by the following equation

| \| $G_{C,j,z} =G_{C,z}^{max}\cdot\frac{\varphi_{z,j}\left[ B_{C,j} \right]}{\sum_{j=1}^{J} \varphi_{z,j}\left[ B_{C,j} \right]+ K_{p}} \left( 1- e^{\Lambda.\sum_{j=1}^{J} \varphi_{z,j}\left[ B_{C,j} \right]} \right)\gamma_{T}$ \| \| --- \| | (S10) |
| --- | --- | --- |

where $G_{C,z}^{max}$ is the maximum grazing rate, *K_p_* the half-saturation constant, *γ_T_* the temperature dependence,$\varphi_{z,j}$ the log preference function

| $\varphi_{z,j}= {\frac{1}{2.\sigma}.e}^{-\frac{{\log\left( \frac{\vartheta}{\vartheta_{opt}} \right)}^{2}}{2.\sigma^{2}}}$ | (S11) |
| --- | --- |

with ϑ the predator:prey biovolume ratio and ϑ*_opt_* its optimal value. The term $\left( 1- e^{\Lambda.\sum_{j=1}^{J} \varphi_{z,j}\left[ B_{C,j} \right]} \right)$ accounts for the decrease of the grazing efficiency for scarce prey populations (i.e., a type III Holling functional response).

- Remineralization and nitrification in the system is taken into account by the term $R_{i}$. For details, see Ward et al (2012)

Depth (m)

Temperature (C°)

-mld-

C biomass (µmol.m^-3^)

P

Z

b.

a.

Fig. S1: a. Temperature depth profile over one year. Black line: the mixed layer depth (mld, the depth at which the temperature is more than 10% lower than at the surface). b. Surface dynamics of total biomass of phytoplankton (P) and zooplankton (Z). The bloom corresponds to the shallower level of mld during spring.


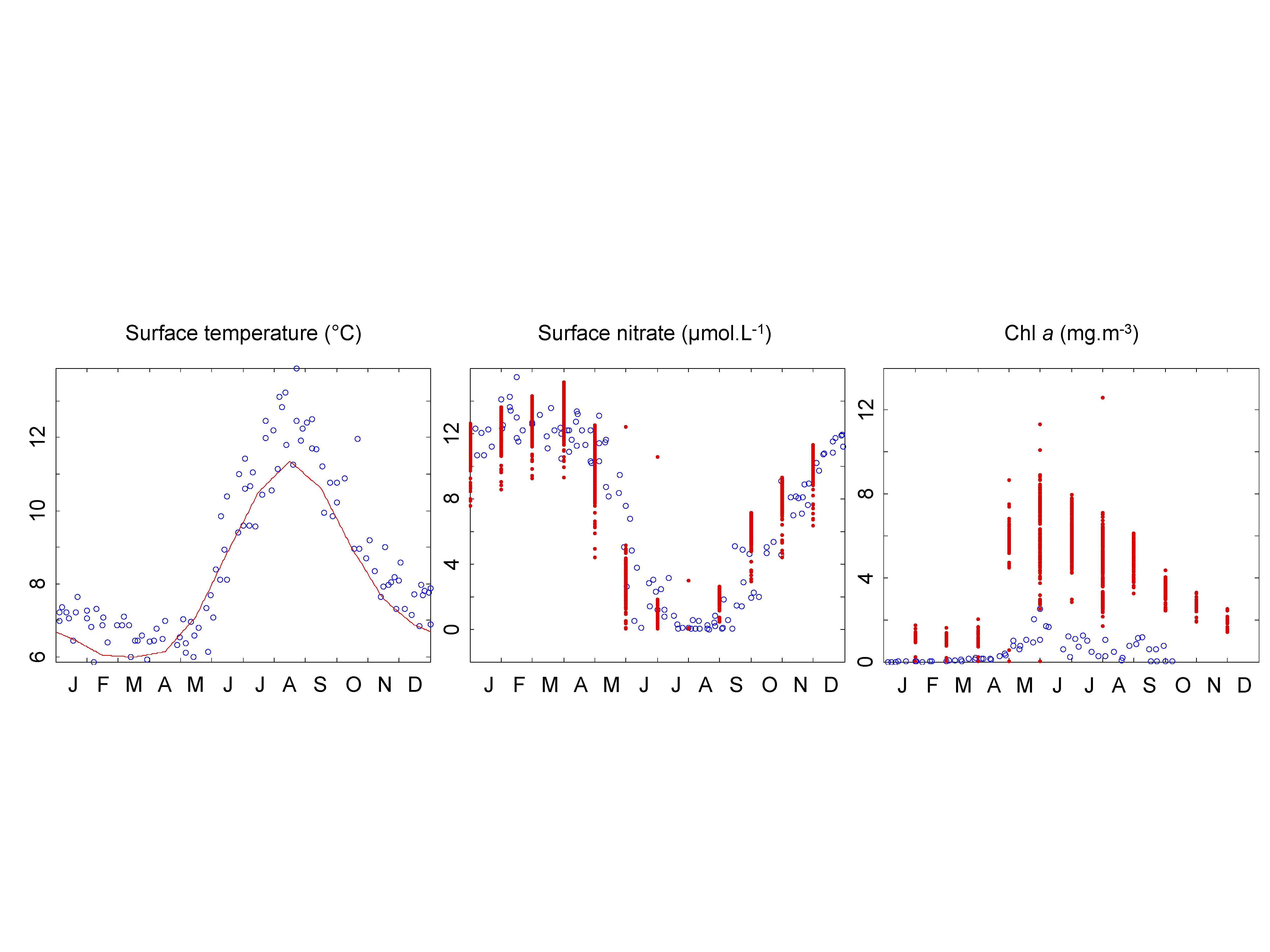


Fig. S2: Annual surface temperature, nitrate and Chlorophyll concentrations. Blue open circles: measurement made in the Norwegian Sea (Dale et. al 1999). Red line and red dots: data extracted from a 350 years simulation with our model (all years are shown).

**Supplementary material 3: *Test of the evolutionary algorithm’s assumptions***

In this section we test the validity of the main assumptions underlying our evolutionary algorithm. The main assumptions are:

(a) a mutant’s relative depth profile converges quickly to a stable distribution such that invasion fitness can be computed by considering population dynamics over a single year;

(b) following replacement of the old residents by the successful mutants the system converges quickly (within two years) to the attractor corresponding to the new set of resident trait values;

(c) “invasion implies replacement” during directional selection. This assumption allows us to skip the phase of explicit competition between a successful mutant and the resident.

We test these assumptions using the model as defined in our ms (main text and Appendix 2), except that we define a reduced ecological community, consisting of a single phytoplankton species (a *Synechococcus*) and a single zooplankton species with a fixed body size. This simpler configuration allows us to obtain more detailed information about the invasion process and to evaluate our assumptions more rigorously.

We first study the consequences of relaxing assumptions (a) and (b), relaxing assumption (c) in the following section.

*The effect of assumptions (a) and (b)*

In order to understand the overall ecological dynamics of this system, we first determine how the (periodically) steady state depends on the phytoplankton trait value (cell volume *x_r_*) in the range from 1 to 41 µm^3^. We assume that the zooplankton (Z) have a body size of $2\times{10}^{4}$ µm^3^ such that their optimal prey size is *x_r_* =2 µm^3^. The simulation results are shown in Fig A1a. This bifurcation plot can be divided into three regions. In **region I** (trait value *x_r_* <2 µm^3^), the phytoplankton population (P) is limited by grazing by the zooplankton population (Z) and P decreases with *x_r_*. The strength of the top-down regulation is maximal for a phytoplankton cell size equal to the optimal prey size of the grazer (2 µm^3^) leading to a minimum in P. At this point, nitrate consumption is hence minimal and N is correspondingly maximal. In **region II** (from 2 µm^3^ to ~ 10 µm^3^), where the phytoplankton cell size is higher than the grazer’s optimal prey size, P increases with the trait value, while N decreases, and Z increases. The minimum of P at *x_r_* =2 µm^3^ can be interpreted as an example of the « pessimization » principle (Mylius and Diekmann 1995) in that optimization of grazing results in pessimization of the grazer’s environment in the sense that its resource will settle at the lowest possible density. In **region III** (from ~10 µm^3^ to 41 µm^3^) the low grazing efficiency due to the large phytoplankton cell size results in a rapid decrease of the zooplankton population and its extinction beyond ~10 µm^3^. Beyond this point the phytoplankton population is not controlled by grazing and reaches a carrying capacity determined by bottom-up regulation. The level of surface nitrate shows a slight increase with the phytoplankton cell size, reflecting that *N** increases with cell size (Figure 1).


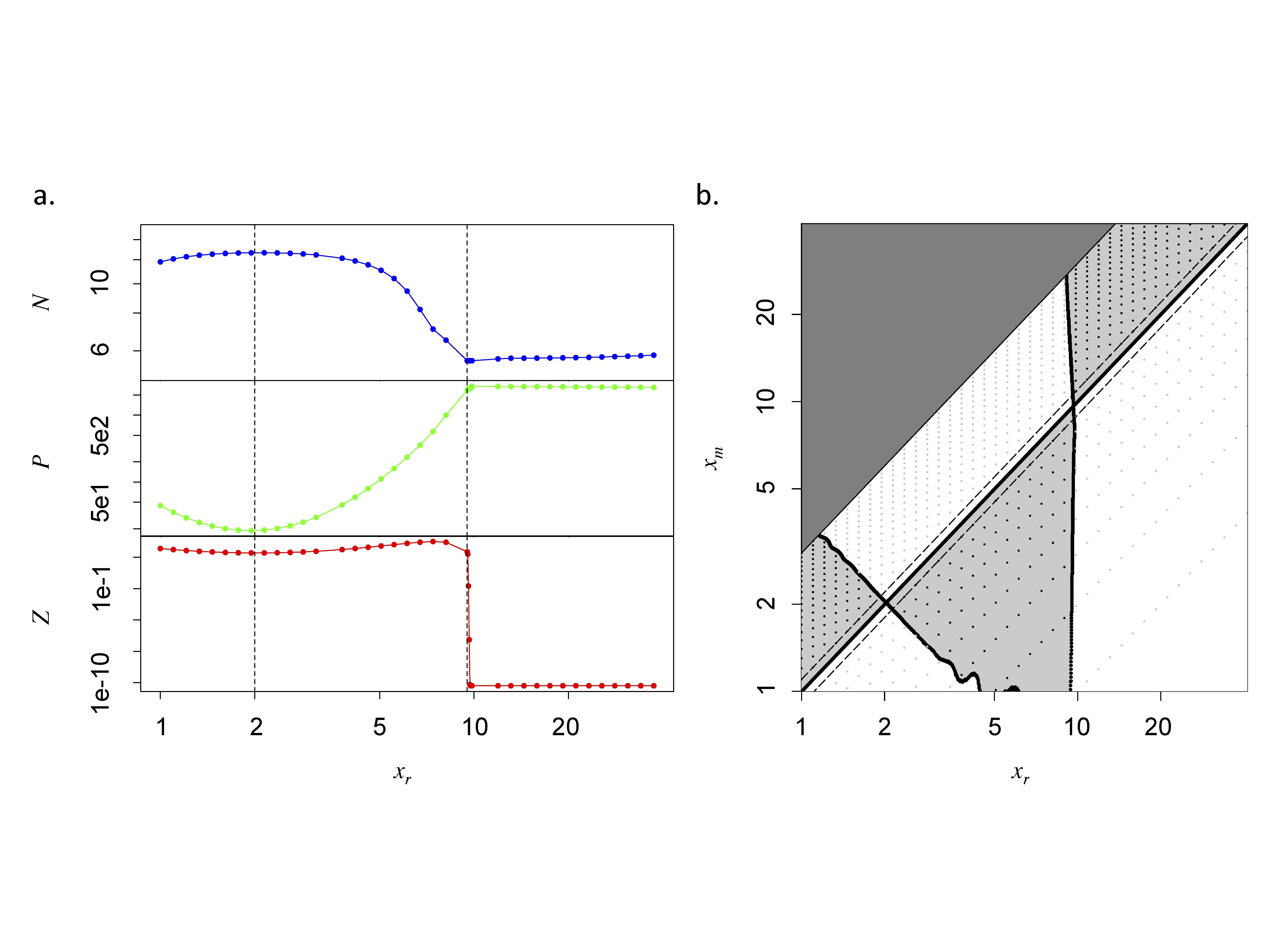


Fig. S3: a. The periodic attractor characterised by three ecological variables averaged over the year, depending on phytoplankton trait value. *N*: nitrate surface concentration in mg.L^-1^, *P*: phytoplankton and *Z*: zooplankton carbon biomass in mmol. b. Pairwise Invasibility Plot obtained for a period of fitness evaluation of 9 years and an ecological transient of 10 years. White regions: positive invasion fitness. Light grey regions: negative invasion fitness. Dark grey regions: no data. Black thick lines: null-clines (zero fitness). Intersections of isoclines are referred as singular points. Dots correspond to the points in which the invasion fitness is actually computed; fitness between points is determined by spline interpolation. Black dashed lines: the mutation step size used in the main study. Note that the singular points in b. are depicted in a. by the two vertical dashed lines, defining regions I, II and III.

We evaluate the effect of relaxing assumptions (a) and (b) by studying their effect on the evolutionary dynamics using the so-called pairwise invasibility plot (PIP), a representation widely used in adaptive dynamics (Geritz et al 1997). Fig A3b shows a PIP corresponding to the bifurcation diagram in Fig A3a. It depicts the sign of invasion fitness of possible mutants for a range of resident strategies (Fig A3b). To obtain this kind of plots we use the same algorithm as used in the main study. Here, however, 40 mutants are introduced instead of two in the main study, each next mutant being 10% smaller or bigger than the previous one.


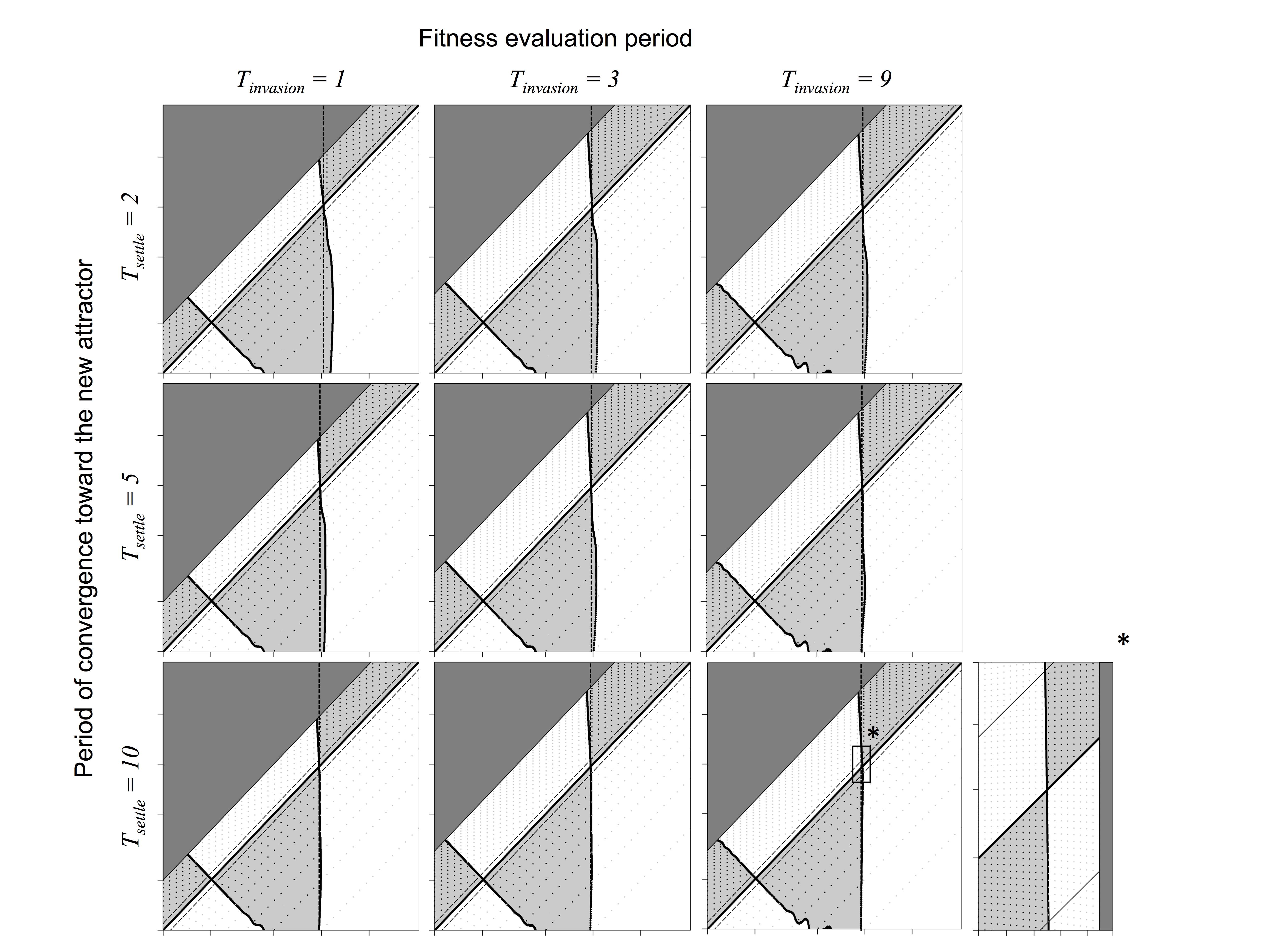


Fig. S4: Pairwise Invasibility Plot for different durations of the period of convergence toward a new attractor (in ordinate) and of the period of fitness evaluation (in abscise). For the maximal conditions, a zoom is performed around the second singularity (black frame) with a better resolution.

We test the effect of two parameters of the evolutionary algorithm (*T*_invasion_ and *T*_settle_) in a 3x3 factorial design. We vary *T*_invasion_ (the duration of the period over which we estimate invasion fitness as the mutant’s exponential growth rate) between one, three and ten years. We vary *T*_settle_ (the duration of the ecological transient, i.e., the time we let the new resident populations to settle on the new attractor) between two, five and nine years. Among these nine configurations, the total number of years between two mutational events therefore varies from three to 19 years.

Fig A3b shows the PIP for the maximum values of these parameters. Any intersection of the diagonal isocline with the second isocline corresponds to a singular point. The PIP thus shows two singular points, which are depicted in Fig A3a by vertical dashed lines, separating the three regions. The right-hand singular point (*x_r_* ~10 µm^3^) is a CSS: it is both convergence stable and evolutionarily stable. Directional selection in the neighbourhood of this CSS will lead to evolution towards this point, which is an evolutionary end-point. The left-hand singular point (*x_r_* = 2 µm^3^) is an evolutionary repeller: directional selection in the neighbourhood of this point leads to evolution away from this point. Note that selection at the singular point is disruptive.

Fig A4 shows the PIPs for the nine combinations of T_invasion_ and T_settle_. It shows that *(i)* the two parameters affect neither the direction of selection, nor the nature of the two singular points, and *(ii)* that in almost all cases, changing the mutation step size would not modify the sign of the invasion fitness. The only exceptions occur for very large mutation step sizes.

*The effect of assumption (c)*

In this section we test the assumption that “invasion implies replacement” by explicitly simulating the competition dynamics between invader and resident following successful invasion by a mutant. Starting at the system’s attractor, we introduce two mutants (one 10% bigger, one 10% smaller) at low initial density. The initial relative depth profile of the mutant is uniformly low ($6\times{10}^{-9}$ mmol.m^-3^). In our simulations we observe systematically that mutants that are predicted to replace the resident according to the PIP (i.e., positive invasion fitness) do indeed invade and eventually outcompete the resident population (Fig A5). The transient, from introduction of the invaders until one of them reaches its attractor and outcompetes the former resident, ranges from a bit more than 2 years to almost 200 years. Our evolutionary algorithm thus correctly identifies the direction of selection and shortens the simulations considerably by skipping the transient of the mutant toward its relative stable depth profile and the whole replacement transient.

In summary, we have shown that the assumptions (a), (b) and (c) are valid for our system using a simplified ecological community. Relaxing any of these assumptions does not modify the predicted evolutionary dynamics. While the full system studied in the study is more complex due to a higher number of species, the dynamics are governed by the same basic principles and hence we argue that the assumptions are met also for the full system.


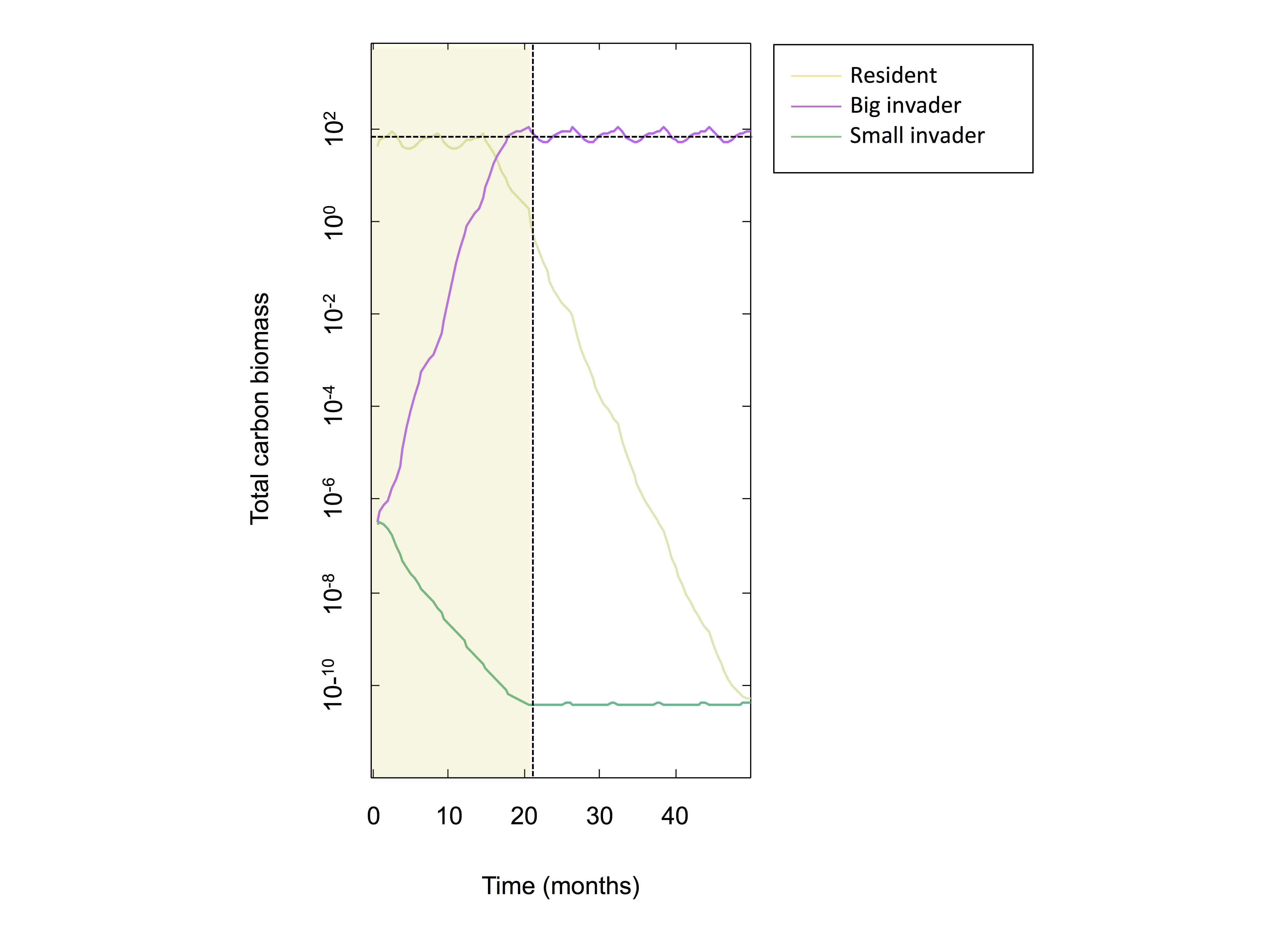

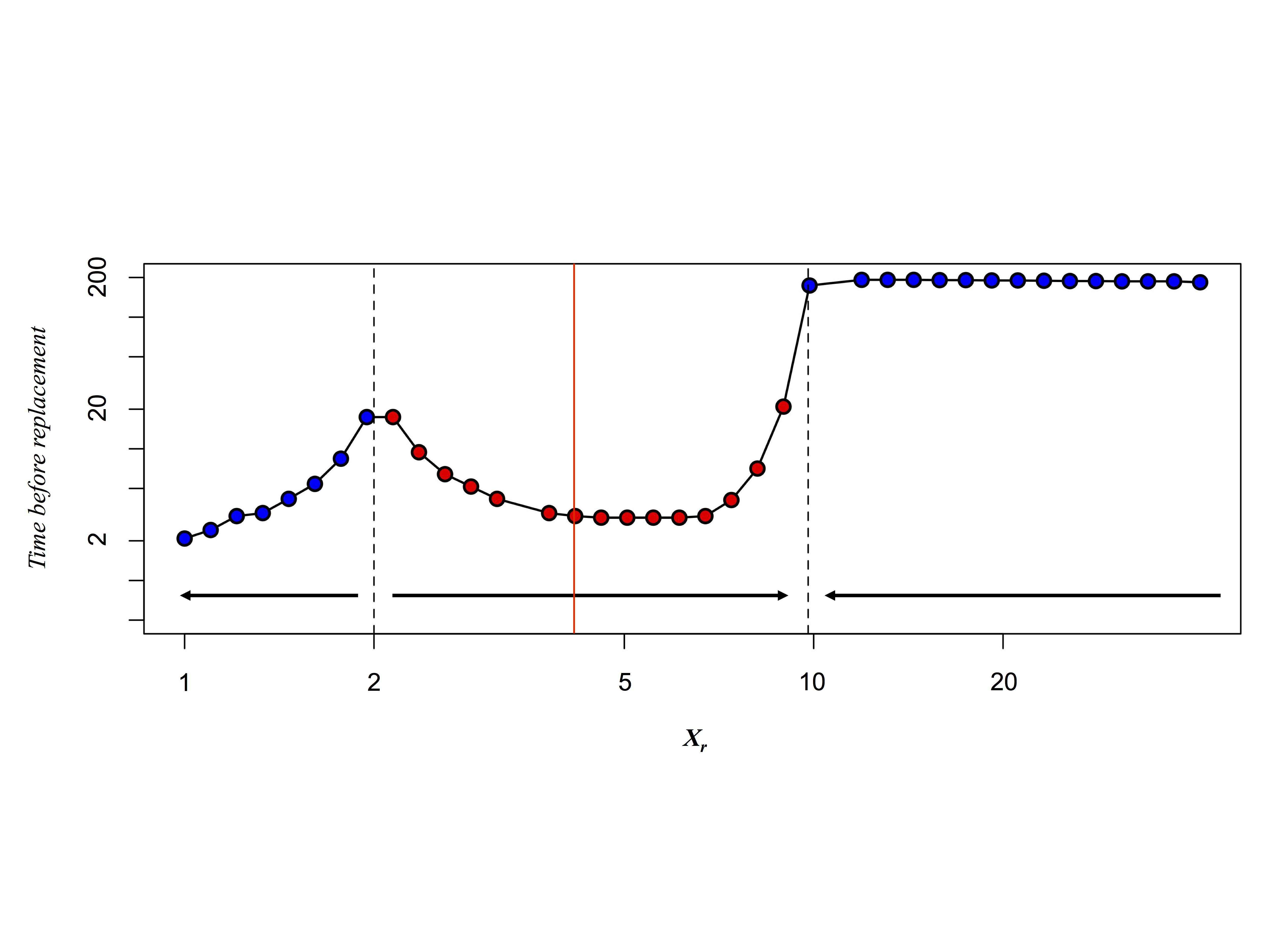


Fig. S5: a. Time required for the successful mutant to replace the resident through competitive exclusion (in years). Blue dots: the smaller mutant replaces the resident. Red dots: the bigger mutant replaces the resident. The arrows show the direction of the selection. The dashed lines correspond to the singular points identified in the PIPs. The red line indicates the particular value of *x_r_* in the simulation showed in b. The yellow area designates the replacement transient. It is evaluated by looking at when the successful mutant (here the bigger one) has reached its limit cycle represented here by the horizontal dashed line. Note that the full extinction is not necessarily required for the former resident to stop affecting the population dynamics of the new one.

a.

b.

Fig. S6. Characteristics of the final state of the ecological phase (upper panel) and the eco-evolutionary phase (lower panel), for the simulations starting with 80 randomly seeded phytoplankton species. Histograms: the mean size distribution of the phytoplankton community averaged over all simulations. Dashed curves: the grazing background averaged over all simulations.


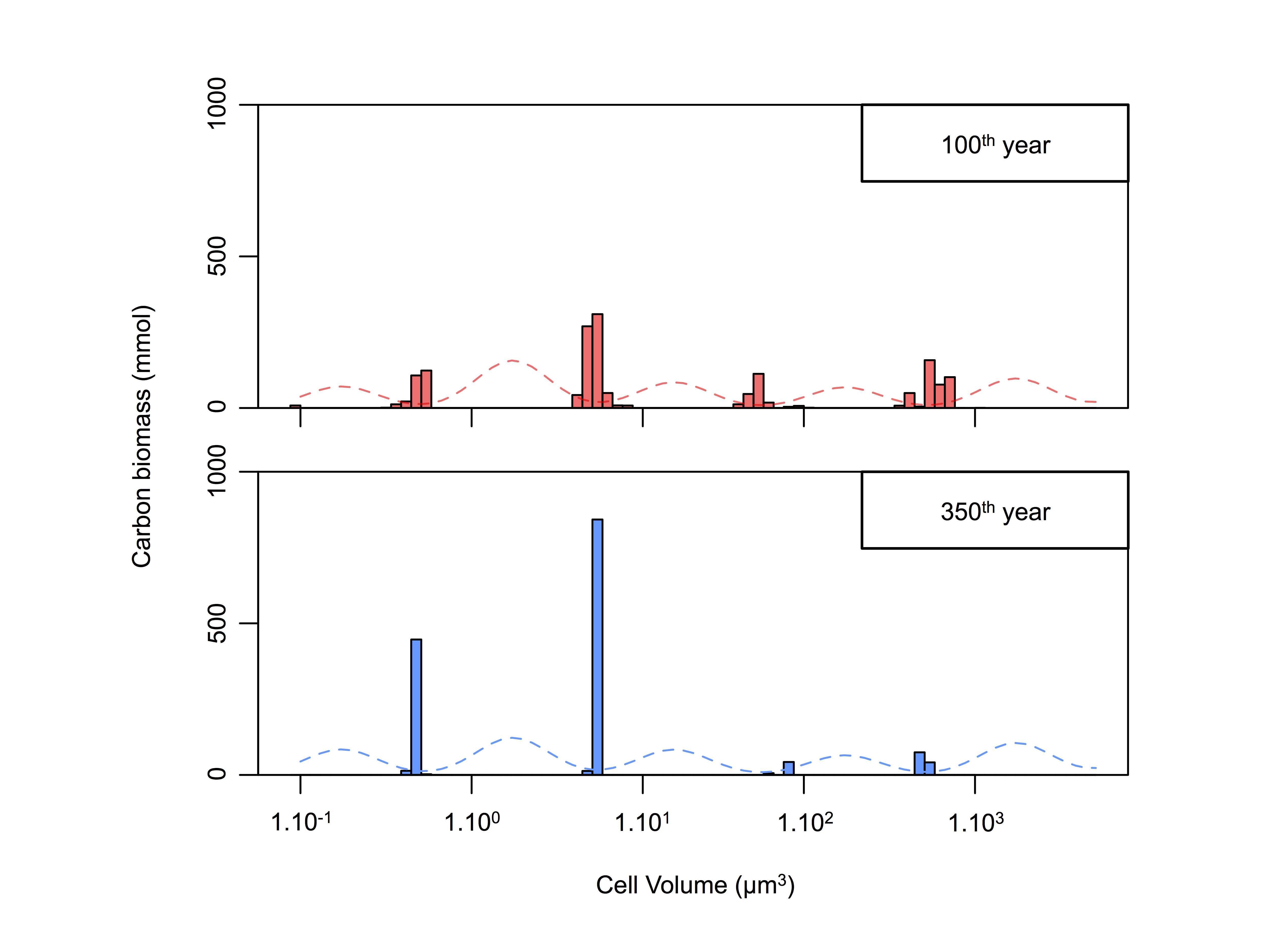


a.

b.

c.

Fig. S7: a. Dynamics of the dominant phytoplankton populations during the last 15 year period at the end of the two phases of the example run (cf Figure 4). From t=85 to 100 years, the dynamics are annually periodic. From 335 to 350 years, the dynamics follow an annual cycle superimposed on a 6-year cycle due to adaptation. b. Zooplankton dynamics during the same periods. c. Trait dynamics of the dominant phytoplankton species from t=335 to 350 years: 6-year alternations between two trait values drive the six-year cycles observed in a and b.
